# Photocaged Hoechst Enables Subnuclear Visualization and Cell Selective Staining of DNA *in vivo*

**DOI:** 10.1002/cbic.202000465

**Published:** 2020-11-02

**Authors:** Carina A. Lämmle, Adam Varady, Thorsten G. Müller, Caterina Sturtzel, Michael Riepl, Bettina Mathes, Jenny Eichhorst, Anje Sporbert, Martin Lehmann, Hans‐Georg Kräusslich, Martin Distel, Johannes Broichhagen

**Affiliations:** ^1^ Department of Chemical Biology Max Planck Institute for Medical Research Jahnstr. 29 69120 Heidelberg Germany; ^2^ St. Anna Children's Cancer Research Institute, Innovative Cancer Models Zimmermannplatz 10 1090 Vienna Austria; ^3^ Department of Infectious Diseases, Virology University Hospital Heidelberg Im Neuenheimer Feld 344 69120 Heidelberg Germany; ^4^ Zebrafish Platform Austria for preclinical drug screening (ZANDR) Zimmermannplatz 10 1090 Vienna Austria; ^5^ Department of Molecular Pharmacology and Cell Biology Leibniz-Forschungsinstitut für Molekulare Pharmakologie (FMP) Robert-Rössle-Straße 10 13125 Berlin Germany; ^6^ Advanced Light Microscopy Max Delbrück Centrum for Molecular Medicine Berlin in the Helmholtz Association Robert-Rössle-Straße 10 13125 Berlin Germany; ^7^ Department of Chemical Biology Leibniz-Forschungsinstitut für Molekulare Pharmakologie (FMP) Robert-Rössle-Straße 10 13125 Berlin Germany

**Keywords:** DNA, Hoechst, labeling, microscopy, zebrafish

## Abstract

Selective targeting of DNA by means of fluorescent labeling has become a mainstay in the life sciences. While genetic engineering serves as a powerful technique and allows the visualization of nucleic acid by using DNA‐targeting fluorescent fusion proteins in a cell‐type‐ and subcellular‐specific manner, it relies on the introduction of foreign genes. On the other hand, DNA‐binding small fluorescent molecules can be used without genetic engineering, but they are not spatially restricted. Herein, we report a photocaged version of the DNA dye Hoechst33342 (pcHoechst), which can be uncaged by using UV to blue light for the selective staining of chromosomal DNA in subnuclear regions of live cells. Expanding its application to a vertebrate model organism, we demonstrate uncaging in epithelial cells and short‐term cell tracking *in vivo* in zebrafish. We envision pcHoechst as a valuable tool for targeting and interrogating DNA with precise spatiotemporal resolution in living cells and wild‐type organisms.

## Graphical abstract

**Precision without genetic engineering**: pcHoechst allows light‐triggered DNA staining by photochemical uncaging. As such, it can be used to visualize and track nuclei with cellular resolution, which is demonstrated in live cells and *in vivo*. This chemical tool does not rely on genetic engineering and can ultimately be used to target and track DNA in subnuclear regions with high resolution.

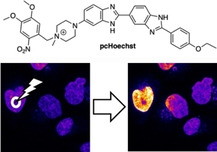

## Introduction

DNA stores the unique genetic code of all organisms.^[1]^ With its iconic double helix structure elucidated by Watson and Crick, it has been one of the most studied biomolecules in the life sciences. As such, many methods are available to mark and investigate DNA by means of fluorescent microscopy.[^2^, ^3^, ^4^] Genetic engineering can be employed to target DNA by using recombinantly expressed fluorescent proteins fused to DNA binders, such as histones,[^5^, ^6^, ^7^] zinc fingers[^8^, ^9^] and Cas9.[^10^, ^11^, ^12^, ^13^] On the other hand, fluorogenic small molecules can be used that either intercalate or bind to the grooves.^[14]^ Both approaches bear advantages and disadvantages. While genetic engineering allows subcellular targeting, it needs the introduction of foreign genes. Small molecules avoid the introduction of exogenous genetic material but lack cellular specificity.

**Figure 1:**
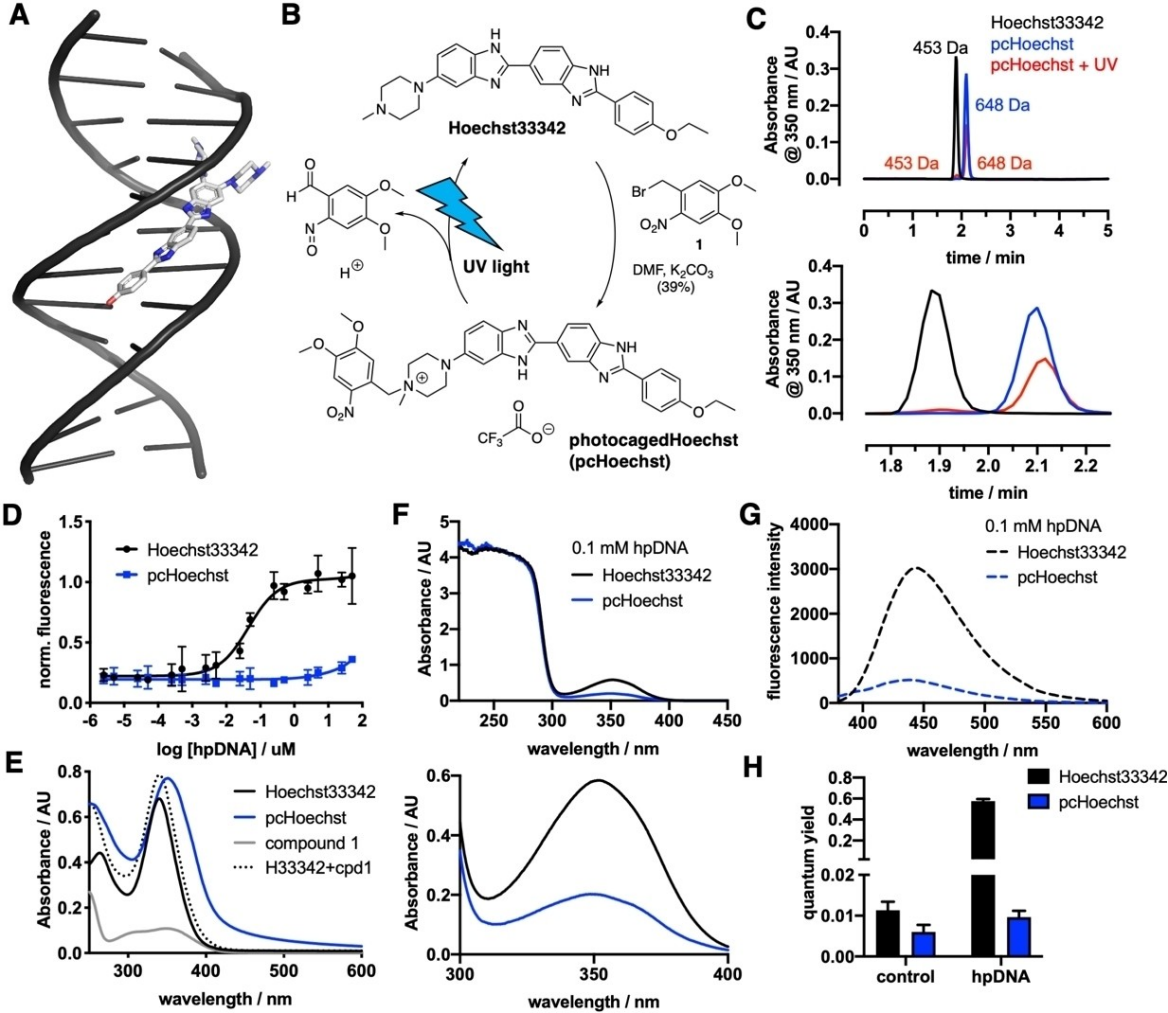
Synthesis and *in vitro* characterization of pcHoechst. A) Hoechst33258 binds to the minor groove of DNA (PDB ID: 8BNA), showing a disordered, partly solvent‐exposed piperazine. B) pcHoechst is obtained by benzylation of Hoechst33342 with an *o*‐nitro benzyl photocage that can be removed with UV light to re‐obtain Hoechst33342. C) Chromatograms of Hoechst33342, pcHoechst and a sample of pcHoechst that was irradiated for 5 min with white light show that the appearance of Hoechst33342 (top) matches the occurrence of Hoechst after pcHoechst irradiation by retention time and molecular weight (enlargement below). D) Concentration‐response curve for Hoechst33342 and pcHoechst against hairpin (hp) DNA. No fluorescence can be detected for pcHoechst. E) UV/Vis spectra of Hoechst33342, pcHoechst and *o*‐nitrobenzylbromide **1**. Mathematical addition of Hoechst33342 plus compound **1** is drawn as a dashed line. F) UV/Vis spectra of Hoechst33342 and pcHoechst in the presence of 0.1 mM hpDNA (top) with enlargement (bottom). G) Fluorescence emission spectra of Hoechst33342 and pcHoechst in the presence of 0.1 mM hpDNA. H) Quantum yield measurements of Hoechst33342 and pcHoechst in the absence (control) and presence of 0.1 mM hpDNA.

The first 2,6‐bis‐benzimidazol derivatives were synthesized in 1974 as chemotherapeutic agents with their fluorescence increase upon binding to DNA already recognized in the initial publication.^[15]^ While these compounds, widely known as “Hoechst”, did not hold promise for use in cancer therapy, they have become an invaluable tool to stain DNA in live cells. Hoechst typifies a chemical biology probe with remarkable characteristics, possessing good permeability, high affinity (*K*_d_∼1–10 nM) and specificity, and a fluorescent turn‐on of ∼30‐ to 40‐fold upon DNA binding with a large 100 nm Stokes shift,^[16]^ when they bind to adenine‐rich regions in the minor groove of DNA (Figure 1a).^[17]^ While these features are favorable, Hoechst is soluble and freely diffusible and will label all DNA present in the tissue being exposed to. Thus, Hoechst cannot be used for targeting selected nuclei of cells within a tissue or even specific chromosomes or DNA regions within a cell. To overcome such limitations, chemical caging of functional groups has been introduced, which relies on masking a crucial binding moiety with a group that can be removed selectively with a bio‐orthogonal reagent. For instance, fluorophores can be liberated by enzymatic removal of caging groups,[^18^, ^19^, ^20^] although this is difficult to target to subcellular or suborganellar regions. Therefore, we chose a caging group that can be liberated by light, which offers unmatched spatiotemporal precision, ease of application, tissue penetration, and relative low toxicity as actuator.^[21]^ In this study, we designed and synthesized photocaged Hoechst (pcHoechst), which stains DNA specifically in targeted HeLa cells after UV to blue‐light‐mediated uncaging. Expanding on this, we explore the utility of pcHoechst *in vivo*, targeting and tracking nuclei of single cells in zebrafish embryos. Ultimately, we showcase subnuclear staining and concomitant tracking of small DNA regions in live mammalian cells.

## Results

With our aim to stain DNA of selected single cells, we installed a photocage on Hoechst33342. This was achieved by reacting free‐based Hoechst33342 with *o*‐nitrobenzyl bromide **1** to give photocaged Hoechst (pcHoechst, Figure 1b, Scheme S1 in the Supporting Information). By running the reaction in DMF with K_2_CO_3_ as a base at 60 °C, we isolated pcHoechst in a satisfying 39 % yield among starting material and bis‐benzylated Hoechst after HPLC purification. Purity was assessed by UHPLC to be >95 % (Figure S1). Benzylation was determined to first occur at the 4‐*N*‐methyl piperazino group to give a quaternary ammonium salt, as determined by ROESY (Figure S2). Illumination with UV to blue light will cleave the molecule, liberating Hoechst33342 (Figure 1c).

In a first *in vitro* experiment to assess fluorescence upon target binding, hairpin DNA (hpDNA) was titrated against 100 nM pcHoechst (blue) and no increase in fluorescence was detected up to 100 μM hpDNA (Figure 1d). Higher DNA concentrations resulted in more intense fluorescence (Figure S3), while Hoechst33342 (100 nM, black) served as a positive control with an EC_50_=44.6 nM. Comparing UV/Vis spectra in PBS (20 μM of compound), we observed an increase and slight red‐shift in maximal absorbance of pcHoechst (blue, *λ*_max_=351 nm) compared to Hoechst33342 (black, *λ*_max_=340 nm; Figure 1e). UV/Vis spectra of compound **1** (gray) under the same conditions revealed *λ*_max_=350 nm, and after calculated addition of the UV/Vis spectra of compound **1** and Hoechst (dashed line) we found that the theoretical spectrum differs from pcHoechst. The extinction coefficient of compound **1** at 350 nm was determined to *ϵ*_350 nm_(**1**)∼5200 M^−1^ cm^−1^ (Figure S4). Comparing fluorescence values at low DNA concentration, we could not observe statistically significant differences between Hoechst and pcHoechst, arguing that quenching of bisbenzimide background fluorescence by the nitrobenzyl group does not occur. However, when absorbance measurements were repeated in the presence of 1 mM hpDNA, we observed a marked increase in absorbance (Figure 1f) and emission (Figure 1g) for Hoechst33342 *versus* pcHoechst. We furthermore estimated turn‐on by increase in fluorescence quantum yield upon hpDNA incubation, increasing by 51‐fold and 1.6‐fold for Hoechst33342 and pcHoechst, respectively (Figure 1h).

**Figure 2:**
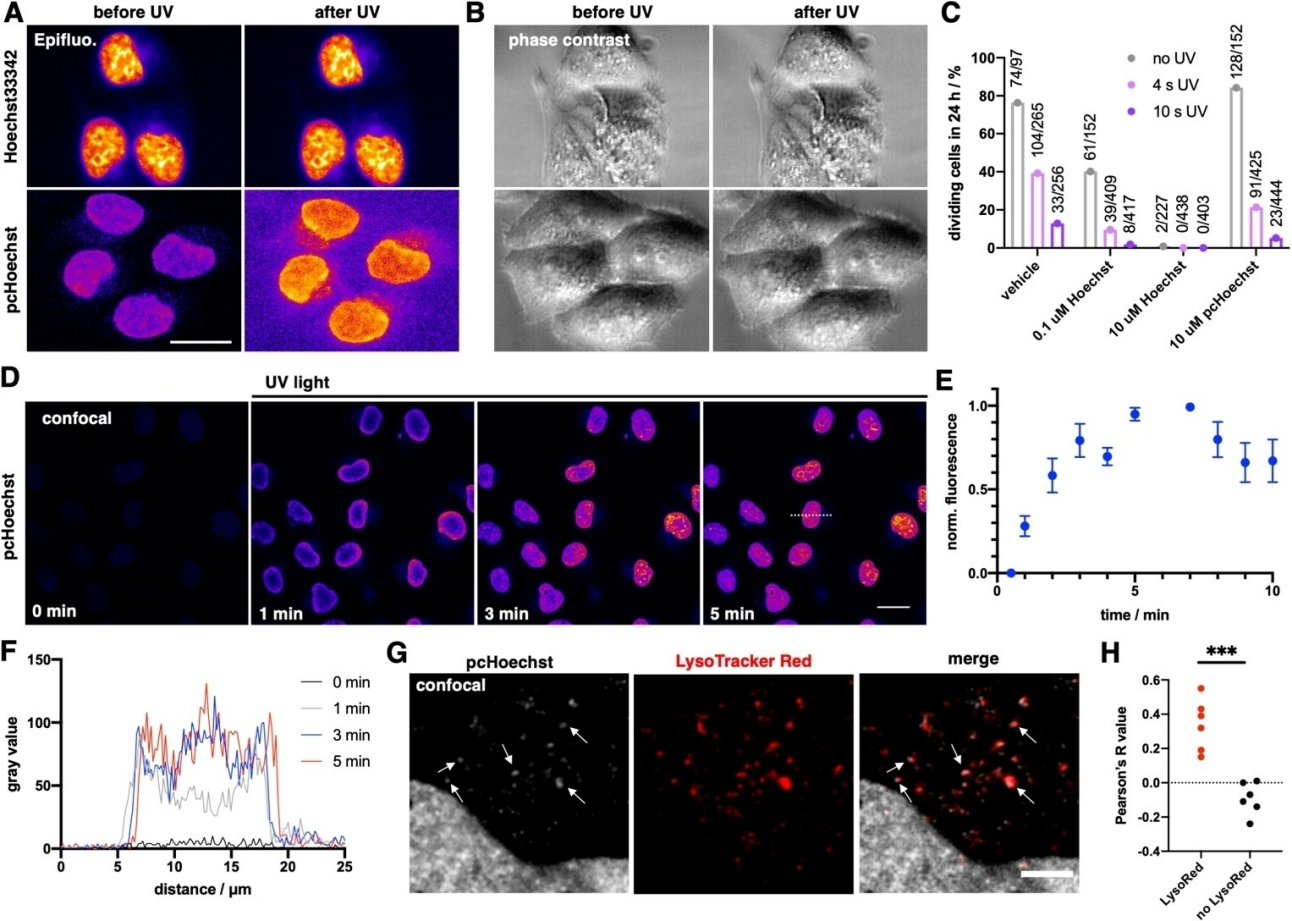
*In cellulo* uncaging of pcHoechst. A) Epifluorescence imaging of live HeLa cells incubated with Hoechst33342 or pcHoechst (10 μM each) before and after 4 s of UV light irradiation shows an increase in nuclear fluorescence for pcHoechst‐treated cells. Scale bar: 20 μm. B) As in (A), but phase contrast images. C) Cells from (A) were imaged over 24 h to determine single cell viability to progress through a complete cell cycle during compound and UV treatment, numbers indicate absolute cell division events. D) Confocal images of live HeLa cells incubated with pcHoechst (10 μM). Between each image, UV light was applied with higher intensity to uncage pcHoechst. Scale bar: 20 μm. E) Fluorescence increase over time upon pcHoechst uncaging (*n*=15 cells). F) Line scans result in fluorescence increase over time upon pcHoechst uncaging (from dashed line in D). G) Colocalization of cytosolic signals from pcHoechst with the lysosomal system labeled with LysoTracker Red. Scale bar: 5 μm. H) Pearson's correlation *R* value for cytosolic colocalization is positive for co‐applied pcHoechst and LysoTracker Red, and negative in controls, thus arguing for extranuclear signals stemming from acidic compartments; *n*=6 cells.

We next turned to imaging experiments *in cellulo* by incubating HeLa cells with Hoechst33342 and pcHoechst (each 10 μM) and illuminated or not with a UV LED (*λ*_uncage_=365 nm, 4 s, 76 μW LED power) to observe an increase in nuclear and background signal in pcHoechst treated cells (Figure 2a, Supporting Movies 1–4). After illumination with UV light, we continued imaging in widefield mode (Figure 2b), and, after 24 hours evaluated the video for how many cells underwent division (Figure 2c). While lethality was high for 10 μM Hoechst33342 even in the absence of UV illumination, it was less pronounced when using 100 nM. Interestingly, pcHoechst showed no toxicity when compared to vehicle treated cells in the absence of UV illumination. For all conditions, UV irradiation showed a clear trend in toxicity, but cell division rate was higher after illumination of cells treated with 10 μM pcHoechst compared to cells treated with 100 nM Hoechst33342. With this initial characterization, we were eager to use pcHoechst with confocal microscopy that offers several advantages: i) shorter pixelwise illumination, ii) improved signal over background, and iii) targeted uncaging is feasible. Again, we started with live HeLa cells to perform first global uncaging of after treatment with 10 μM pcHoechst. As such, an immediate increase in fluorescence was observed after applying a high frequency UV light scan with high intensity (*λ*_uncage_=355 nm, 15 s, 1.10 mW laser power) before image acquisition (*λ*_ex_=355 nm, *λ*_em_=420–500 nm), reaching saturation within 5 minutes (Figure 2d, e, Supporting Movie 5). Longer imaging decreased fluorescence in nuclear zones, presumably reflecting bleaching, which is supported by using even higher laser powers (Figure S5). Analysis of the fluorescence intensity of line profiles over time indicate initial signal appearance in heterochromatin‐rich regions at the nuclear lamina within one minute after uncaging, followed by an increase inside the nucleus within 3 minutes after uncaging (Figure 2f). This might reflect the fact that condensed heterochromatic regions are stained preferentially, which is known for Hoechst derivatives,^[22]^ or that abundant cytoplasmic uncaged Hoechst diffuses into the nucleus and stains the regions it comes into contact with first. Spots outside of the nucleus were visible that we assign to accumulation in the lysosomal system. This is supported by imaging in the presence of mCLING,^[23]^ a stain for the detection of the endo‐ and lysosomal system, and LysoTracker red, a stain for acidified lysosomes, which both show co‐localization with uncaged pcHoechst with no apparent bleedthrough (Figures 2g, S6, and S7) and with a positive Pearson's coefficient *R*=0.338±0.151 (Figure 2h, *R*=−0.260±0.115 for images without LysoTracker). We further quantified the extranuclear signal to be 7.2±3.2 % of the total integrated signal density. Interestingly, and further support for our observation of lysosomal signal, Hoechst33342 and pcHoechst display slightly different spectra and higher quantum yields in citric acid buffer (pH 4; Figure S8).

We next investigated if pcHoechst can be applied in living animals to specifically stain and track single nuclei. As such, we first examined pcHoechst staining of nuclei in zebrafish cells *in vivo* and compared its performance to Hoechst33342. Zebrafish embryos were incubated in pcHoechst (100 μM) or Hoechst33342 (100 nM, 1 μM, 10 μM and 100 μM) for 24 hours. 100 nM and 1 μM Hoechst33342 were well tolerated, at 10 μM Hoechst33342 survival was slightly reduced, whereas 100 μM Hoechst33342 was lethal and 10 μM Hoechst33342 still showed some toxicity. In contrast, 100 μM pcHoechst did not affect zebrafish development when fish were kept under ambient light or in the dark (Figure S9). Five minutes′ UV irradiation using 395 nm UV‐LEDs of 100 μM pcHoechst‐treated zebrafish embryos did also not show any toxic effects (Figure S9), likely due to inefficient uncaging yielding concentrations lower than 10 μM of active compound. Still, we observed that this UV irradiation successfully uncaged pcHoechst as several nuclei became visible, indicating that pcHoechst can be used in zebrafish embryos. Fish kept in the dark or under ambient light did not show any Hoechst‐labeled structures, demonstrating that pcHoechst is not leaky under these conditions (Figure S10).

**Figure 3:**
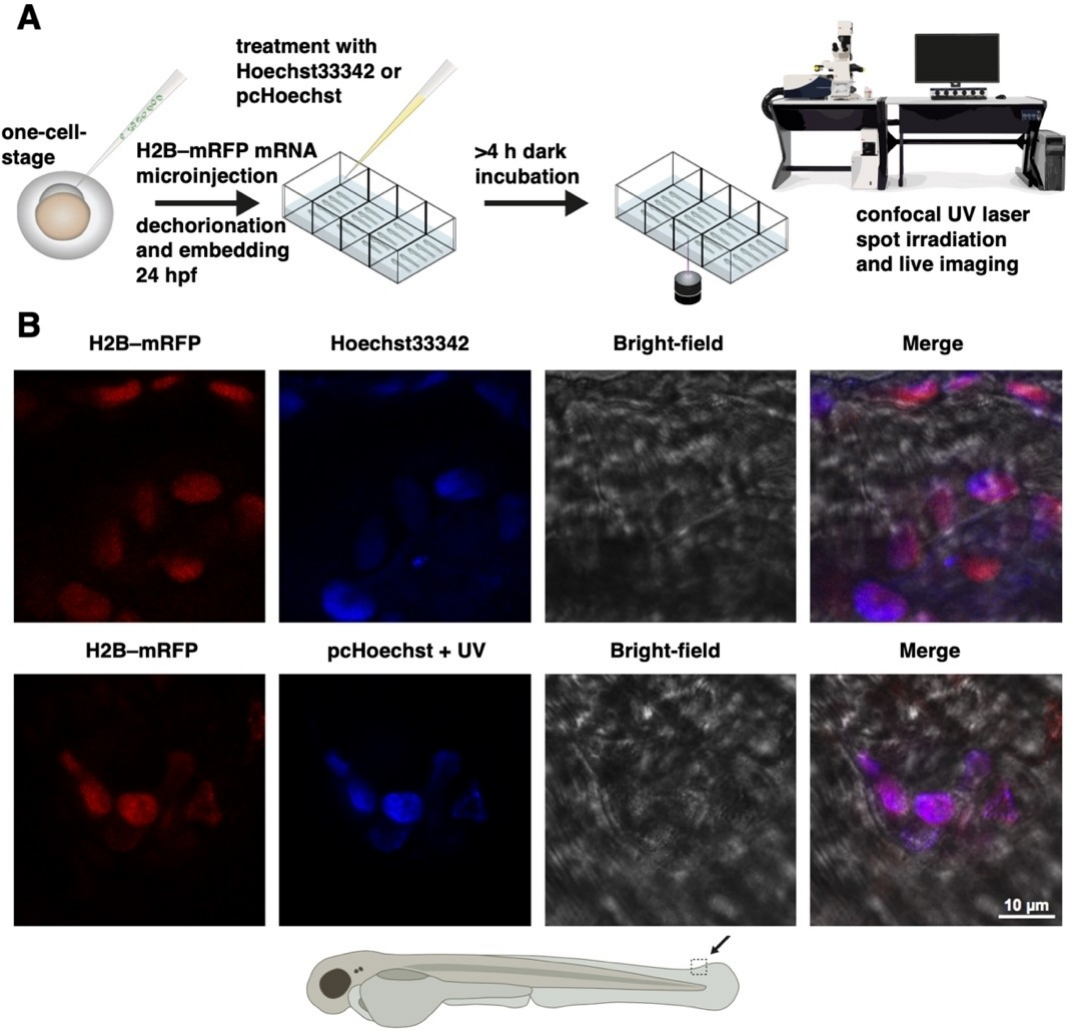
*In vivo* imaging of cell nuclei in zebrafish with pcHoechst. A) General schematic workflow for experiments in Figures 3, 4, S11, and S12. H2B‐RFP mRNA is injected into single‐cell‐stage zebrafish embryos for correlative nuclear staining of pcHoechst with a red fluorescent DNA‐associated fusion protein (Histone 2B fused to mRFP). Hoechst33342 or pcHoechst is applied (between 32–56 hpf), with 4–16 hours′ incubation in the dark before targeted uncaging and subsequent imaging. B) Agarose‐embedded H2B‐RFP‐expressing zebrafish embryos were incubated with 10 μM Hoechst 33342 (upper row) or 100 μM pcHoechst (lower row) overnight at 32 hpf. Spot irradiation using a 405 nm UV laser targeted at a large cluster of cells was performed for uncaging pcHoechst at 52 hpf. Confocal imaging of the targeted area at the zebrafish tail fin at 54 hpf shows colocalization of pcHoechst (blue, bottom row) with mRFP‐tagged H2B (red) in comparison to Hoechst33342 (blue, top row). Merge includes bright‐field image. The shown images are representative of three uncaging experiments using the same parameters.

To demonstrate that the pcHoechst‐labeled structures in zebrafish are indeed nuclei, we used an mRFP‐tagged histone protein H2B (H2B‐mRFP) as nuclear reference stain (Figure 3a). H2B‐mRFP expressing embryos were dechorionated and incubated in 10 μM Hoechst33342 or 100 μM pcHoechst starting at 32 hpf. At 52 hpf pcHoechst was activated by employing localized 405 nm UV laser illumination on a confocal microscope targeting a cluster of cells. Like Hoechst33342, pcHoechst stained nuclei of zebrafish cells and colocalized with H2B‐mRFP after UV illumination, indicating that pcHoechst does bind to DNA after uncaging (Figure 3b). Again, no fluorescent pcHoechst signal was detected in zebrafish embryos when handled under ambient light and without UV illumination, indicating that pcHoechst is not very light‐sensitive. We observed staining of nuclei only in superficial cell layers with both Hoechst33342 and pcHoechst and therefore continued to specifically target superficial cells in the following experiments.

**Figure 4:**
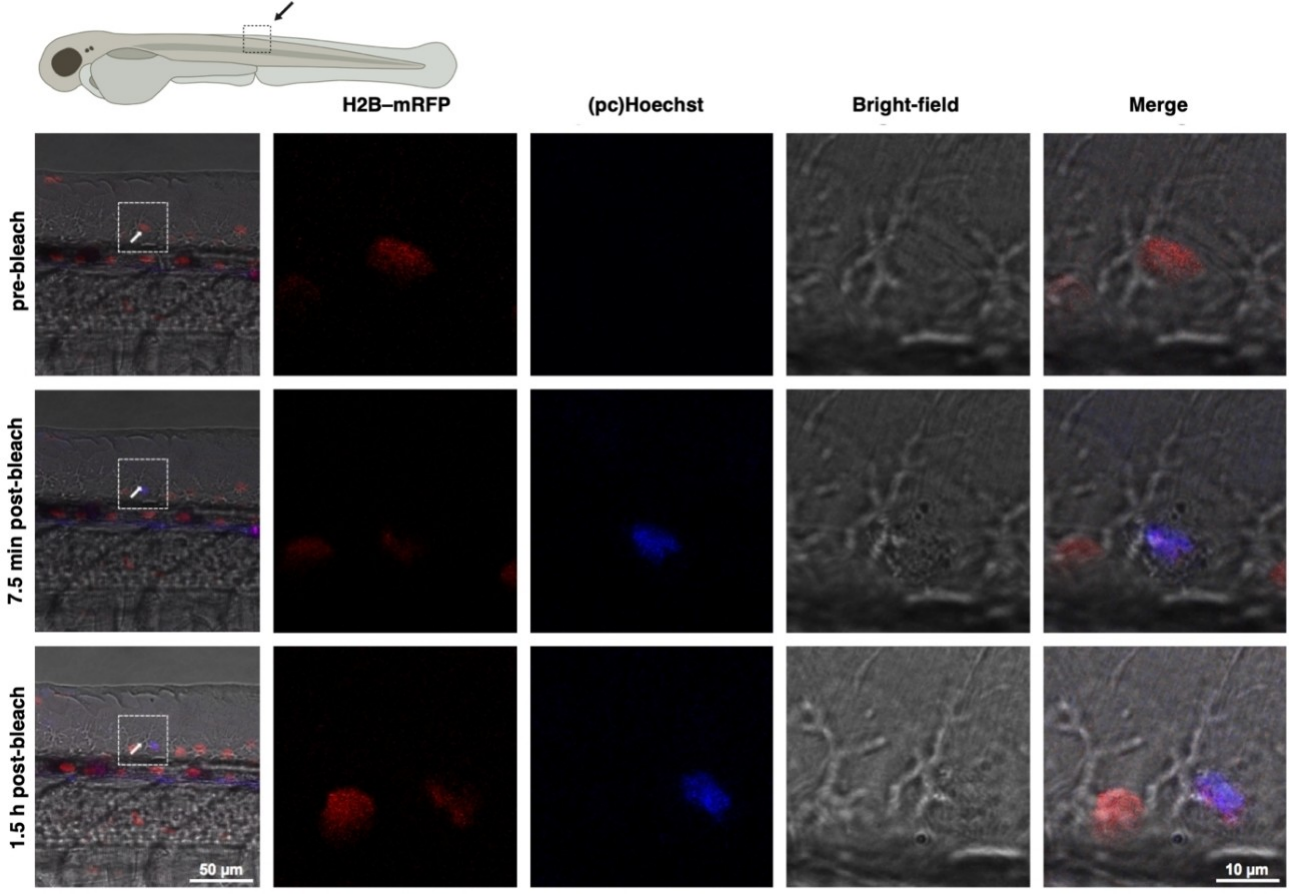
*In vivo* uncaging of pcHoechst with optimized light intensity at single‐cell resolution. H2B‐mRFP (red) injected zebrafish embryos were incubated with 100 μM pcHoechst in the dark overnight and activated by using a 405 nm UV laser at 0.07 mW and at 74 hpf. The arrow indicates a nucleus targeted with two bleach points. A pre‐bleach image was recorded prior to illumination, post bleach images 7.5 min and 1.5 h after bleaching. The white rectangles depict magnified areas. Uncaged pcHoechst (blue) can be observed 7.5 min and 1.5 h after UV illumination and colocalizes with H2B‐mRFP (red) in a single nucleus. Representative images of two experiments with the same intensity, and ten experiments in total.

To establish spatially controlled activation of pcHoechst *in vivo*, we employed the bleach‐point function of a confocal microscope to target single cells in zebrafish embryos. Illumination was performed using 100 cycles of 100 ms light pulses of a 405 nm UV laser. To identify the optimal laser power required to locally uncage pcHoechst in zebrafish with minimal UV‐inflicted damage, we tested a range of laser powers from 0.0175 to 0.24 mW. At high powers (0.20–0.24 mW) staining of nuclei in the proximity of targeted cells could be observed, but fluorescence of mRFP‐tagged H2B was bleached and the targeted region at the tail of the embryo was noticeably injured (Figure S11). In this setting, pcHoechst signal was not observed directly in the targeted nuclei, most likely due to death of the targeted cells or tissue movements due to injury. Further reduction of the UV laser power to 0.07 mW activated pcHoechst at single‐cell resolution, retained the H2B‐mRFP signal of targeted cells and generally attenuated any damage inflicted on targeted cells (Figure 4). Scanning of the UV laser at low power (0.0175 mW) for imaging did not activate pcHoechst in any cells, indicating a threshold intensity between 0.0175 and 0.07 mW for pcHoechst activation in our setup. To investigate whether activated pcHoechst can be used for cell tracking, we performed time‐lapse recording after uncaging of pcHoechst with 0.07 mW for up to 1.5 hours (Supporting Movie 6). We observed that the stained nucleus maintained pcHoechst signal over this time period. Additional experiments revealed that pcHoechst stained nuclei are visible for at least 10 hours post illumination (Figure S12), demonstrating that tracking of selected cells is possible with pcHoechst for several hours.

**Figure 5:**
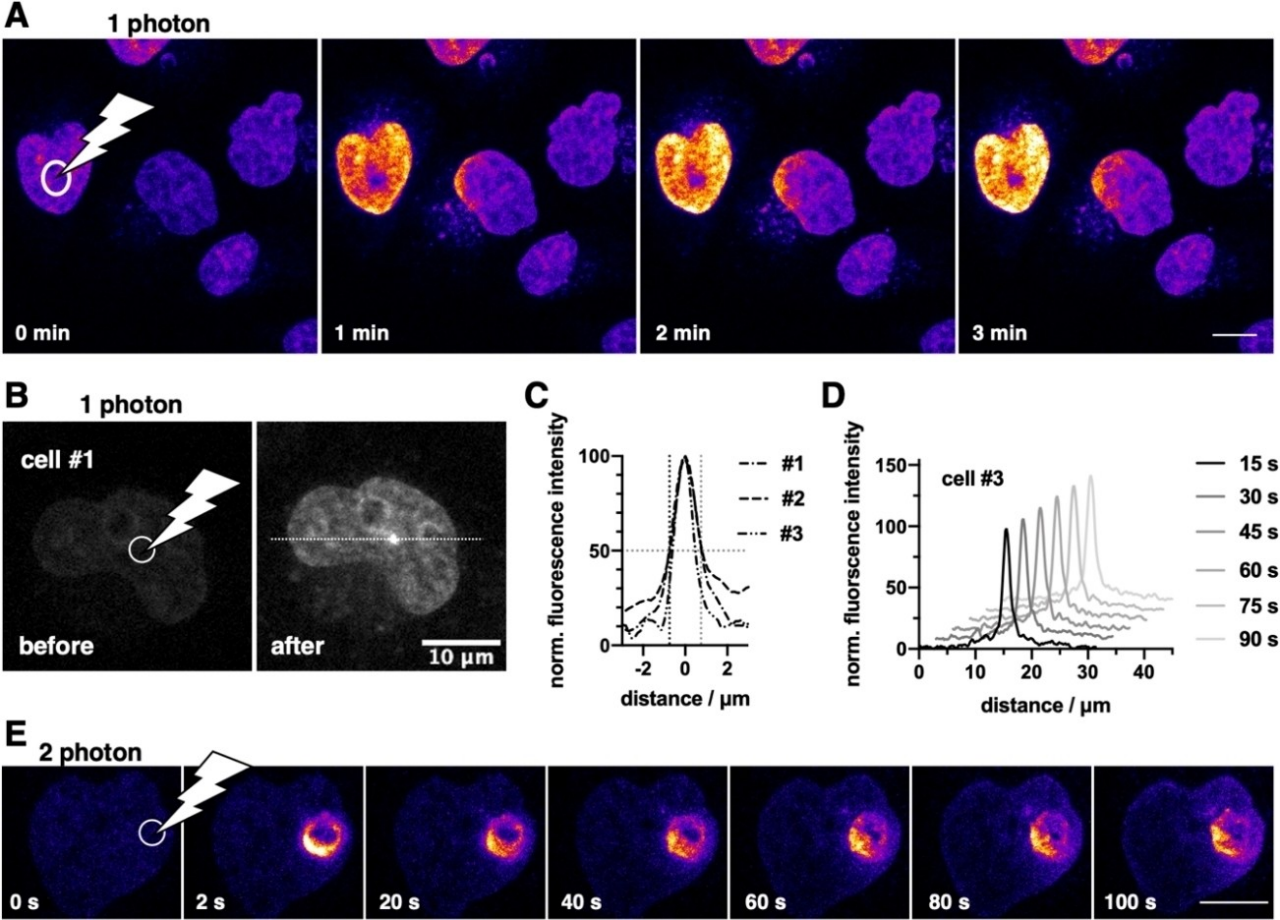
pcHoechst uncaging allows cell targeting and subnuclear visualization of DNA. A) Confocal imaging of live HeLa cells incubated with pcHoechst (100 nM) using a FRAP bleach point with UV light in the indicated area (white circle) shows nuclear staining. High laser power (110–1100 μW for 10–15 s) activates the entire cell. Scale bar: 10 μm. B) As for (A) but with lower laser power (1.42 μW for 10–15 s) allows more precise uncaging in a fraction of the nucleus C) with a resolution of 1.4–1.7 μm that D) remained stable over 90 s. Representative images from three repetitions. E) Confocal images of live HeLa cells incubated with pcHoechst (10 μM) targeting a bleach point with a two‐photon laser (720 nm, 19.7 mW) in the indicated area (white circle) shows subnuclear staining in the illuminated periphery. Representative images of *n*>15 cells. Scale bar: 10 μm.

Next, we wondered if we can gain even finer localization of DNA with pcHoechst, enabling subnuclear staining. To prove this, we turned again to HeLa cells, which allow an easier setup than zebrafish. By using a bleach‐point function in a fluorescence recovery after photobleaching (FRAP) experiment, we were able to uncage pcHoechst with cellular specificity, similar to zebrafish. To accomplish this, we added 100 nM pcHoechst to live HeLa cells prior to imaging, and applied local UV illumination (*λ*_uncage_=355 nm, 10–15 s, 0.11–1.10 mW) to observe fluorescence immediately afterwards (*λ*_ex_=355 nm, *λ*_em_=420–500 nm) mainly in the nucleus of the targeted cell (Figure 5a). A dark spot in the targeted region can be noticed after applying UV light, which we attribute to Hoechst bleaching, as demonstrated in live cells (Figure S13). Encouraged by this, we aimed to push this further by staining DNA spatiotemporally precise in a smaller, subnuclear area. By decreasing the laser intensity in our setup (*λ*_uncage_=355 nm, 10–15 s, 1.42 μW) on a local defined spot we were able to stain DNA with uncaged Hoechst with a resolution down to 1.4 μm (Figure 5b, c), paving the way for fine‐tuned chromosomal staining and observation. Intriguingly, we were able to observe the corresponding signal over 90 s without observing significant signal broadening, allowing short‐term tracking of DNA in a subnuclear fraction (Figure 5d).

Ultimately, and in order to reduce phototoxicity, we aimed to use two‐photon excitation for uncaging of pcHoechst. As wavelengths are in the NIR region, two‐photon microscopy allows for deeper tissue penetration with reduced scattering, background and phototoxicity outside of the focal plane, however, higher laser intensities are needed.^[24]^ As such, we incubated live HeLa cells with 10 μM pcHoechst for 3 hours and then transferring the cells to an upright laser scanning confocal microscope equipped with a tunable NLO laser for two‐photon excitation. Unfortunately, we were not able to globally uncage, yet were able to bleach the dim nuclear signal of pcHoechst with low intensities (*λ=7*20 nm, 5.6 mW; Supporting Movie 7). We therefore performed the uncaging and imaging with high and low laser powers (*λ*_uncage_=720 nm, 19.7 mW, *λ*_image_=720 nm, 2.5 mW), respectively, which led to bleaching in the targeted region. Nevertheless, liberated fluorescent signals in the periphery of the bleach point that spread over time demonstrated that two‐photon uncaging is feasible (Figure 5e).

Staining DNA has been at the forefront of molecular biology and four major approaches can be distinguished: i) genetic engineering, where DNA‐binding proteins, such as histones, zinc fingers and Cas9, are fused to fluorescent proteins, ii) dye‐tagged oligonucleotides, as used for FISH and DNA‐PAINT, iii) the use of small‐molecule DNA binders, and iv) metabolic labeling of DNA, where unnatural nucleotides are incorporated during DNA synthesis and afterwards reacted in a bio‐orthogonal way *via* click chemistry. While all these techniques are powerful in their own way, we sought to expand this palette with an additional tool, which can be used *in vivo* with subcellular precision and without the need to create a transgenic animal. These desirable characteristics limit the design to DNA‐targeting small‐molecule probes, of which a substantial amount has been developed in the past: ranging from Hoechst33342 itself, fluorescent dyes have been chemically fused to the bisbenzimide scaffold to shift spectra into the visible^[25]^ and to enable super‐resolution imaging.[^22^, ^26^] However, these dyes stain DNA globally, so we had to find a way to activate our probe on demand. In the past, molecular stains have been developed that are using light to become able to bind to DNA, such as styrylbenzothiazole^[27]^ derivatives and thiazole orange modified diarylethenes,^[28]^ and therefore may amend to the spatiotemporal precision that illumination allows. However, styrylbenzothiazoles need UV‐C light (220 nm) to be converted into their DNA binding counterpart and therefore seem to be incompatible with microscopic setups (especially in live cells and animals), while functionalized diarylethenes are large in size and photochromism is slow, and thereby only applicable to fixed cells with long irradiation times (15 min). For these reasons, we synthesized pcHoechst, a cell‐permeable, caged DNA stain based on Hoechst33342 that can be uncaged in microscopic studies with UV to blue light (355–405 nm) illumination in live cells. Consulting a crystal structure of bound Hoechst33258 to DNA (PDB ID: 8BNA^[17]^), the 4‐*N*‐methyl piparazino group shows rotamers and is partly solvent exposed (Figure 1a); however, using an *o*‐nitrobenzyl protecting group to form the quaternary ammonium salt renders the molecule unable to turn‐on fluorescence in presence of DNA up to 100 μM *in vitro*. In our assay, the protecting group did not display quenching behavior of the bisbenzimide's background fluorescence. Although we cannot exclude binding of pcHoechst to DNA in our read‐out, we rely on turn‐on fluorescence and not physical binding in our experiments, rendering further investigations of the exact mechanism negligible. While other molecules, such as dyes and drugs are usually protected with cages on functional groups like carboxylates and carbamates to liberate acids and amine, respectively,^[21]^ quaternary ammonium salts were described before as being “photocageable”, such as in a study that used caged nicotine.^[29]^ pcHoechst adds favorably to this report, where we show that tertiary amines are amenable to other photocaging groups. However, we were not able to uncage pcHoechst quantitatively *in vitro* but observed further oxidation products upon prolonged light exposure. This is also reflected in non‐quantitative uncaging *in vivo*, as derived from our toxicity assessment (*vide infra*).

Uncaging pcHoechst in a biological system to mark DNA was first performed in live HeLa cells on a confocal microscope, observing an increase in fluorescence (10 μM pcHoechst, 4 h pre‐incubation) after UV illumination. After acquisition of a first image, uncaging was performed for 1 frame at higher laser power before the next image was taken, allowing the nucleus to be stained globally. These initial experiments highlight the breadth of applications possible with pcHoechst and represents, to the best of our knowledge, the first example of a non‐genetically encoded dye that is able to stain a targeted region within the nuclear organization. Interestingly, the nucleus was stained heterogeneously in the beginning, with signals arising from the outer nuclear zones before becoming homogenous after prolonged exposure. We determined that photoproducts accumulate in the lysosomal system, most probably due to increase in quantum yield in an acidic environment, with the red and far‐red stains LysoTracker red and mCLING, respectively. These control experiments further confirm that using longer wavelengths in conjunction with pcHoechst is feasible, allowing dual‐color microscopy.

A general consideration is the toxicity of small‐molecule DNA binders, especially when they are supposed to be employed over hours in live cells.[^30^, ^31^] Notably, pcHoechst is nontoxic up to 10 and 100 μM for HeLa cells and zebrafish embryos and larvae, respectively, whereas the same concentrations of Hoechst33342 were lethal (first signs of toxicity were observed at 0.1 and 10 μM in cells and in fish, respectively). This argues for no or only weak DNA binding of pcHoechst, and suggests that the active concentration after uncaging *in cellulo* and *in vivo* is below 0.1 and 10 μM, respectively. Furthermore, we determined the toxicity of UV irradiation by flashing HeLa cells for 0, 4 and 10 seconds with a UV LED on an epifluorescence widefield microscope, showing a clear relationship of time and toxicity. As such, UV‐induced DNA phototoxicity,^[32]^ such as photolysis and (dye) crosslinking, needs to be considered. Nevertheless, single cells in which pcHoechst has been uncaged with UV light were still able to complete mitosis and divide.

pcHoechst is taken up by fish cells in a similar manner as Hoechst33342, and both appeared limited to superficial cell layers *in vivo*. Once uncaged, pcHoechst counterstained H2B‐mRFP expressing nuclei *in vivo* with a robust fluorescent signal and remained detectable for multiple hours. With our current illumination protocol for zebrafish embryos, single cells could be labeled and followed over time. However, still some residual UV‐inflicted tissue damage was observed at target sites of successful pcHoechst uncaging. While this may limit applications *in vivo*, further optimization of the irradiation intensity and duration, and or switching to two‐photon activation,^[24]^ might reduce damage inflicted on zebrafish tissues and DNA, and increase the specificity of targeted staining. By using a bleach‐point module, we were able to selectively stain a targeted nucleus with high laser power, or subnuclear regions with reduced laser power. In the first experiment, we observe a dark spot in the targeted region, which we attribute to dye bleaching, and indeed, controls showed that Hoechst33342 was bleached similarly in live HeLa cells. In the second experiment, we highlight subnuclear staining, with selectivity close to the applied light distribution, by using low laser intensities. We furthermore showed that uncaging is possible using the two‐photon effect at *λ*=720 nm in live HeLa cells with high powers using a bleach point. In this setting, we observed staining in the periphery of the bleach spot unlike one‐photon uncaging. This opens avenues for 1 photon uncaging with a pulse of UV to blue light, and subsequent imaging with two‐photon microscopy, enabling milder imaging conditions. While cell and DNA damage by different mechanisms cannot be ruled out, it is important to acknowledge that experimental outcomes with Hoechst dyes and UV‐A irradiation, and as such phototoxicity, may differ between microscopic setups that needs to be evaluated from case to case.[^30^, ^31^, ^33^] For further refinement, the chemical space allows installation of a different photocage with different photophysical properties on the piperazine,[^21^, ^34^, ^35^] whereas conjugation to a fluorescent dye on the phenol offers future possibilities for bathochromic shifts for imaging.[^22^, ^25^, ^26^] Lastly, in our experience, pcHoechst can be handled in lit rooms and imaged with UV lasers at low power without unintended background activation. As such, we highlight pcHoechst as a straightforward chemical biology tool that is used for light‐activated staining of DNA in live cells and zebrafish.

New tools are needed in times where microscopy and nanoscopy are on the forefront to elucidate structures to the nanometer level.^[36]^ pcHoechst enables the global, single cell and subnuclear labeling of DNA in a spatiotemporal manner.

## Conclusion

In summary, we showcase pcHoechst, a photo‐caged DNA stain, and demonstrate its utility for DNA labeling and tracking *in cellulo* and *in vivo*. We envision that pcHoechst and its future derivatives will be useful for visualizing not only chromosomal DNA, but other compartmentalized, extranuclear DNA, which is for instance observed after HIV entry events^[37]^ and in neuroblastoma cancer cells.^[38]^ With the possibility to expand the color palette, this may open up new avenues to interrogate fundamental biological processes, such as tumorigenesis, cell proliferation, development, and viral infections, in native tissue and wild‐type animals.

## Conflict of interest

The authors declare no conflict of interest.

## Supporting information

As a service to our authors and readers, this journal provides supporting information supplied by the authors. Such materials are peer reviewed and may be re‐organized for online delivery, but are not copy‐edited or typeset. Technical support issues arising from supporting information (other than missing files) should be addressed to the authors.

Supplementary

Supplementary

Supplementary

Supplementary

Supplementary

Supplementary

Supplementary

Supplementary
