# Supplementary material for: Photocaged Hoechst Enables Subnuclear Visualization and Cell Selective Staining of DNA in vivo
Source: Chembiochem. 2020 Nov 2;22(3):548–56. doi: 10.1002/cbic.202000465 (PMC7894298; doi:10.1002/cbic.202000465)
Supplement: Supplementary file 1 — Supplementary [file CBIC-22-548-s001.pdf]

ChemBioChem

Supporting Information

Photocaged Hoechst Enables Subnuclear Visualization and Cell Selective Staining of DNA *in vivo*

Carina A. Lämmle[+], Adam Varady[+], Thorsten G. Müller, Caterina Sturtzel, Michael Riepl, Bettina Mathes, Jenny Eichhorst, Anje Sporbert, Martin Lehmann, Hans-Georg Kräusslich, Martin Distel, and Johannes Broichhagen*

Table of Contents

1. General 2

2. Synthesis 3

2.1. Supporting Scheme 1 3

2.2. 1-(Bromomethyl)-4,5-dimethoxy-2-nitrobenzene (1) 3

2.3. 2'-(4-Ethoxyphenyl)-5-(4-methylpiperazin-1-yl)-1H,1'H-2,5'-bibenzo[d]imidazole (free-base Hoechst33342) 3

2.4. 1-(4,5-Dimethoxy-2-nitrobenzyl)-4-(2'-(4-ethoxyphenyl)-1H,1'H-[2,5'-bibenzo[d]imidazol]-6-yl)-1-methylpiperazin-1-ium 2,2,2-trifluoroacetate (pcHoechst x TFA) 4

2.5. 2'-(4-Ethoxyphenyl)-6-(4-methylpiperazin-1-yl)-1H,1'H-2,5'-bibenzo[d]imidazole (Hoechst33342) 4

3. NMR Spectra 5

3.1. 1-(Bromomethyl)-4,5-dimethoxy-2-nitrobenzene (2) 5

3.2. 1-(4,5-Dimethoxy-2-nitrobenzyl)-4-(2'-(4-ethoxyphenyl)-1H,1'H-[2,5'-bibenzo[d]imidazol]-6-yl)-1-methylpiperazin-1-ium 2,2,2-trifluoroacetate (PhotocagedHoechst x TFA) 6

4. Methods 7

4.1. In vitro characterization 7

4.2. Cell culture 7

4.3. HeLa cell experiments 8

4.4. Calculating Cytosolic/nuclear fraction of pcHoechst 9

4.5. Calculating Pearson's R coefficient 9

4.6. Zebrafish strains and maintenance 9

4.7. Determining (pc)Hoechst33342 toxicity and pcHoechst leakiness in zebrafish 9

4.8. Determining uncaging toxicity 10

4.9. mRNA microinjections 11

4.10. In vivo UV laser-based single cell uncaging 11

4.11. Laser power and intensity calculations 12

5. Supplemental Figures 14

6. Supplemental Movies 25

7. Reference 26

1. General

All chemical reagents and anhydrous solvents for synthesis were purchased from commercial suppliers (Sigma-Aldrich, Fluka, Acros, Fluorochem, TCI) and were used without further purification or distillation. If necessary, solvents were degassed either by freeze-pump-thaw or by bubbling N_2 through the vigorously stirred solution for several minutes.

NMR spectra were recorded in deuterated solvents on a Bruker AVANCE III HD 400 equipped with a CryoProbe and calibrated to residual solvent peaks ($^1H/^{13}C$ in ppm): $CDCl_3$ (7.26/77.00), DMSO-d_6 (2.50/39.52). Multiplicities are abbreviated as follows: s = singlet, d = doublet, t = triplet, q = quartet, p = pentet, br = broad, m = multiplet. Coupling constants *J* are reported in Hz. Spectra are reported based on appearance, not on theoretical multiplicities derived from structural information.

LC-MS was performed on a Shimadzu MS2020 connected to a Nexera UHPLC system equipped with a Waters ACQUITY UPLC BEH C18 (1.7 µm, 50 × 2.1 mm). Buffer A: 0.1% FA in H_2O Buffer B: MeCN. The typical gradient was from 10% B for 0.5 min → gradient to 90% B over 4.5 min → 90% B for 0.5 min → gradient to 99% B over 0.5 min with 1 mL/min flow. UPLC-UV/Vis for purity assessment was performed on a Waters H-class instrument equipped with a quaternary solvent manager, a Waters autosampler, a Waters TUV detector and a Waters Acquity QDa detector with an Acquity UPLC BEH C18 1.7 µm, 2.1 × 50 mm RP column (Waters Corp., USA). Buffer A: 0.1% TFA in H_2O Buffer B: 0.1% TFA in MeCN. The typical gradient was from 5% B for 0.5 min → gradient to 95% B over 3.0 min → 95% B for 0.9 min → gradient to 5% B over 1.1 min with 0.6 mL/min flow.

High resolution mass spectrometry was performed using a Bruker maXis II ETD hyphenated with a Shimadzu Nexera system. The instruments were controlled via Brukers otofControl 4.1 and Hystar 4.1 SR2 (4.1.31.1) software. The acquisition rate was set to 3 Hz and the following source parameters were used for positive mode electrospray ionization: End plate offset = 500 V; capillary voltage = 3800 V; nebulizer gas pressure = 45 psi; dry gas flow = 10 L/min; dry temperature = 250 °C. Transfer, quadrupole and collision cell settings are mass range dependent and were fine-adjusted with consideration of the respective analyte's molecular weight. For internal calibration sodium format clusters were used. Samples were desalted via fast liquid chromatography. A Supelco TitanTM C18 UHPLC Column, 1.9 µm, 80 Å pore size, 20 × 2.1 mm and a 2 min gradient from 10 to 98% aqueous MeCN with 0.1% FA (H_2O: Carl Roth GmbH + Co. KG ROTISOLV® Ultra LC-MS; MeCN: Merck KGaA LiChrosolv® Acetonitrile hypergrade for LC-MS; FA - Merck KGaA LiChropur® Formic acid 98%- 100% for LC-MS) was used for separation. Sample dilution in 10% aqueous ACN (hyper grade) and injection volumes were chosen dependent of the analyte's ionization efficiency. Hence, on-column loadings resulted betwee 0.25–5.0 ng. Automated internal re-calibration and data analysis of the recorded spectra were performed with Bruker's DataAnalysis 4.4 SR1 software.

Preparative RP-HPLC was performed on a Waters e2695 system equipped with a 2998 PDA detector for product collection (at 220, or 650 nm) on a Supelco Ascentis® C18 HPLC Column (5 µm, 250 × 21.2 mm). Buffer A: 0.1% TFA in H_2O Buffer B: MeCN. The typical gradient was from 10% B for 5 min → gradient to 90% B over 45 min → 90% B for 5 min → gradient to 99% B over 5 min with 8 mL/min flow.

Flash column chromatography was performed on a Biotage Isolera One with pre-packed silica columns (0.040–0.063 mm, 230-400 mesh, Silicycle). Reactions and chromatography fractions were monitored by thin layer chromatography (TLC) on Merck silica gel 60 F254 glass plates.

2. Synthesis

2.1. Supporting Scheme 1

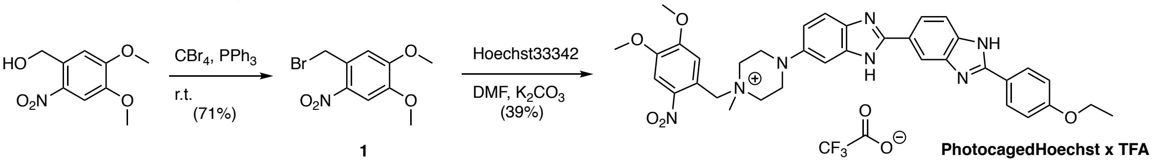

2.2. 1-(Bromomethyl)-4,5-dimethoxy-2-nitrobenzene (1)

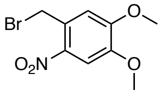

A round bottom flask wrapped with aluminium foil was charged with 144 mg (675 µmol, 1.0 equiv.) of (4,5-dimethoxy-2-nitrophenyl)methanol dissolved in 10 mL DCM, to which 269 mg (811 µmol, 1.2 equiv.) of CBr_4 and 213 mg (811 µmol, 1.2 equiv.) PPh_3 were added in one portion. The reaction mixture was stirred o.n. at r.t. before it was directly subjected to FCC (100% DCM) to obtain 133 mg (482 µmol) of the desired product as a white solid after drying in 71% yield.

^{1}H NMR (400 MHz, $CDCl_3$): δ [ppm] = 7.67 (s, 1H), 6.94 (s, 1H), 4.87 (s, 2H), 4.00 (s, 3H), 3.96 (s, 3H).
^{13}C NMR (101 MHz, $CDCl_3$): δ [ppm] = 153.2, 148.9, 140.3, 127.4, 113.7, 108.5, 56.5, 56.5, 30.1.
HRMS (ESI): calc. for $C_9H_{11}BrNO_4$ $[M+H]^+$: 275.9866 and 277.9846, found: 275.9867 and 277.9849.
R_f (TLC, DCM) = 0.6.

2.3. 2'-(4-Ethoxyphenyl)-5-(4-methylpiperazin-1-yl)-1*H*,1'*H*-2,5'-bibenzo[*d*]imidazole (free-base Hoechst33342)

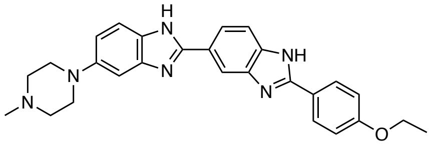

In a 50 mL Falcon tube, 100.0 mg (~178 µmol) Hoechst33342 trihydrochloride (Aldrich, #B2261-100 mg) were dissolved in 20 mL of dH_2O before 240 µL of a 2.3 M K_2CO_3 solution was added. The resulting precipitate was vortexed vigorously and subsequently centrifuged (4,000 rpm, 10 min). The solution was decanted and the remaining residue washed with 5 mL of dH_2O, centrifuged and decanted three times to obtain the free-base as a faint yellow powder after lyophilization, which was used without further purification. This procedure was described previously.[1]

2.4. 1-(4,5-Dimethoxy-2-nitrobenzyl)-4-(2'-(4-ethoxyphenyl)-1*H*,1'*H*-[2,5'-bibenzo[*d*]imidazol]-6-yl)-1-methylpiperazin-1-ium 2,2,2-trifluoroacetate (pcHoechst x TFA)

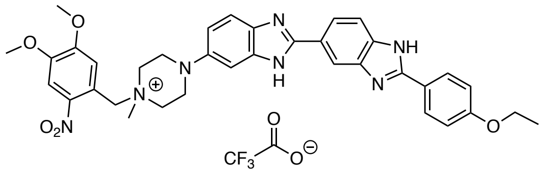

A round bottom flask was charged with 26.0 mg (57.5 µmol, 1.0 equiv.) of free-based Hoechst33342 31.7 mg (230 µmol, 4.0 equiv.) of K_2CO_3 suspended in 2.5 mL DMF, to which 31.8 mg (115 µmol, 2.0 equiv.) of 1-(bromomethyl)-4,5-dimethoxy-2-nitrobenzene was added in one portion. The reaction mixture was stirred o.n. at 60 °C protected from light before it was quenched by addition of 30 µL glacial HOAc and 100 µL water and subjected to RP-HPLC (10-90% MeCN, 60 minutes, λ = 360 nm, T = 30, t_R ~ 27 minutes) to obtain 14.3 mg (22.1 µmol) of the desired product as a faint yellow powder after lyophilization in 39% yield.

^{1}H NMR (400 MHz, DMSO-d$_6$): *δ* [ppm] = 8.46 (d, *J* = 1.7 Hz, 1H), 8.20 (d, *J* = 8.9 Hz, 2H), 8.07 (dd, *J* = 8.5, 1.7 Hz, 1H), 7.89 (d, *J* = 8.5 Hz, 1H), 7.81 (s, 1H), 7.73 (d, *J* = 9.0 Hz, 1H), 7.42 (s, 1H), 7.33 (dd, *J* = 9.1, 2.2 Hz, 1H), 7.26 (d, *J* = 2.2 Hz, 1H), 7.17 (d, *J* = 9.0 Hz, 2H), 5.13 (s, 2H), 4.15 (q, *J* = 7.0 Hz, 2H), 3.98 (s, 3H), 3.94 (s, 3H), 3.90–3.79 (m, 2H), 3.79–3.56 (m, 4H), 3.46 (td, *J* = 11.5, 10.9, 5.6 Hz, 2H), 3.09 (s, 3H), 1.38 (t, *J* = 7.0 Hz, 3H).
^{13}C NMR (101 MHz, DMSO-d$_6$): *δ* [ppm] = 161.0, 158.4 (q, *J* = 34.2 Hz) 154.0, 152.2, 150.0, 149.5, 148.1, 143.6, 141.5 (by HMBC), 141.1 (by HMBC), 138.9 (by HMBC), 134.2 (by HMBC), 129.0, 127.3 (by HMBC), 122.1, 120.4, 118.2, 116.3, 116.3 (q, *J* = 296 Hz), 115.5, 115.1, 114.7, 114.5 (2x by HSQC), 109.3, 99.2, 64.0, 63.5, 59.0, 56.6, 56.4, 44.0, 42.8, 14.6.
HRMS (ESI): calc. for $C_{36}H_{38}N_7O_5$ $[M]^+$: 648.2929, found: 648.2923.

2.5. 2'-(4-Ethoxyphenyl)-6-(4-methylpiperazin-1-yl)-1*H*,1'*H*-2,5'-bibenzo[*d*]imidazole (Hoechst33342)

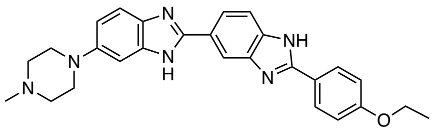

In vitro uncaging was performed by mixing 5 µL pcHoechst (10 mg/mL) with 2.5 µL dH_2O and 2.5 µL PBS in an 1.5 mL Eppendorf tube, which was exposed to white light stemming from a LSE340/1 / 850.27D lamp (LOT Quantum Design), water cooled and coupled to a regulatory unit supplying 445 W. A sample was removed before and one sample 5 minutes after light application and submitted to UHPLC-MS measurements. Hoechst33342 (Aldrich, #B2261) served as reference.

LRMS (ESI): calc. for $C_{36}H_{38}N_7O_5$ $[M]^+$: 648, found: 648 for pcHoechst.
LRMS (ESI): calc. for $C_{27}H_{29}N_6O$ $[M+H]^+$: 453, found: 453 for Hoechst33342

3. NMR Spectra

3.1. 1-(Bromomethyl)-4,5-dimethoxy-2-nitrobenzene (2)

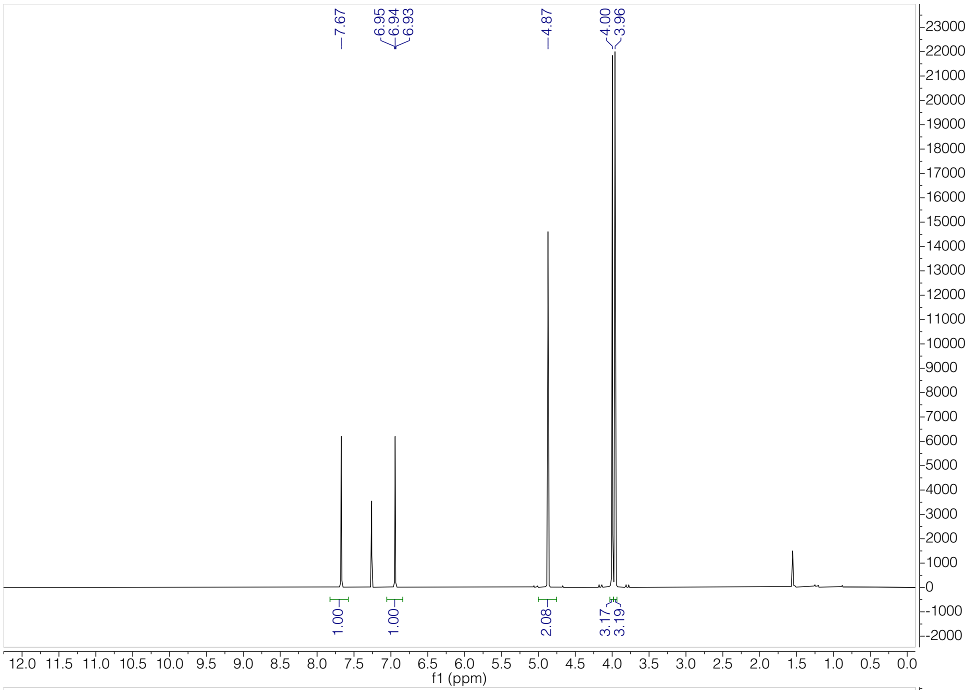

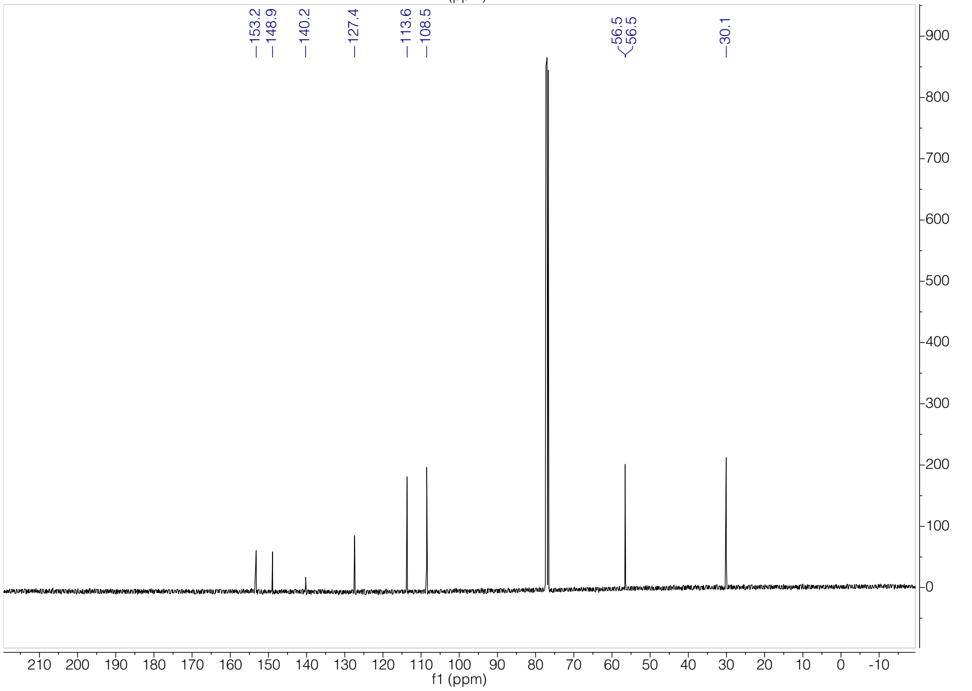

3.2. 1-(4,5-Dimethoxy-2-nitrobenzyl)-4-(2'-(4-ethoxyphenyl)-1*H*,1'*H*-[2,5'-bibenzo[*d*]imidazol]-6-yl)-1-methylpiperazin-1-ium 2,2,2-trifluoroacetate (PhotocagedHoechst x TFA)

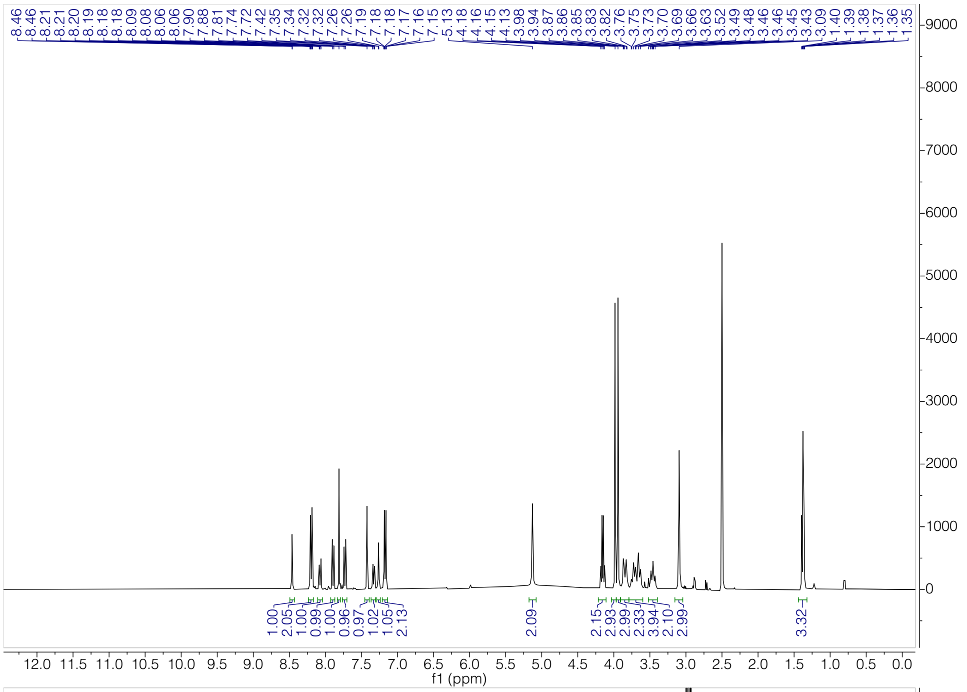

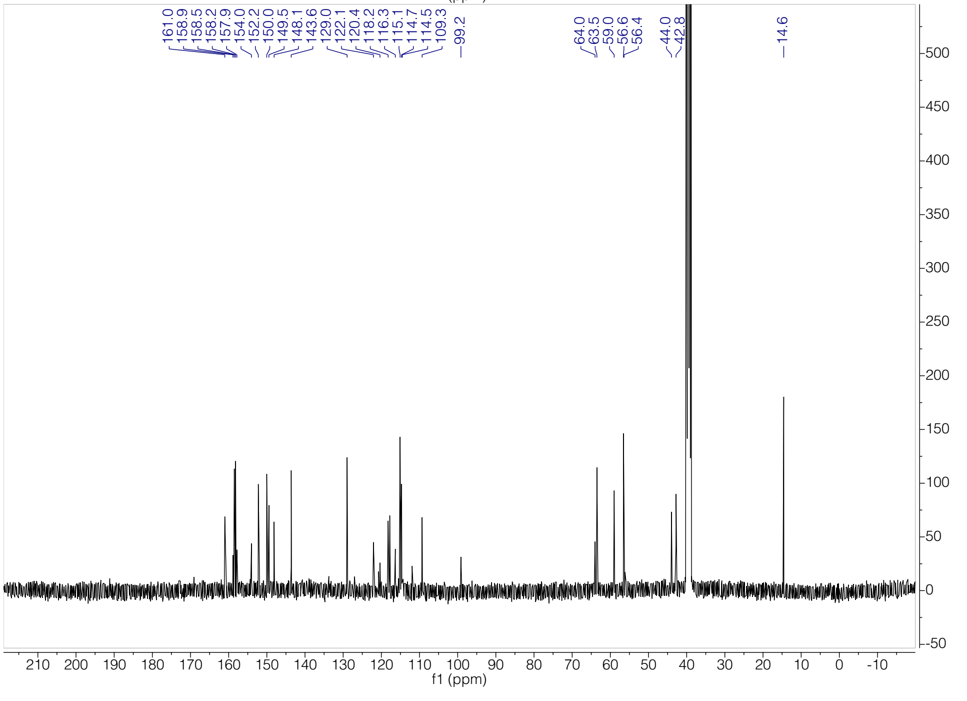

4. Methods

4.1. *In vitro* characterization

hpDNA (5’-CGCGAATTCGCGTTTTCGCGAATTCGCG-3’, Eurofins Scientific SE, saltfree, 2 mM in Milli-Q® water) was heated to 80 ºC for 15 min and allowed to cool slowly to room temperature before it was further diluted in Milli-Q® water. Hoechst33342 and pcHoechst (DMSO stock, 50 µM) were dissolved in PBS pH 7.4 (gibco, 1X, ThermoFisher) with BSA (0.1 mg/mL) and added to the diluted hpDNA to a final concentration of 100 nM. Fluorescence was measured after 1 h incubation at room temperature in 386-well plates (Polystyrene, flat bottom, black, Corning) on a multiwell plate reader Spark® 20M (Tecan) at room temperature exciting at 350 nm (bandwidth = 20 nm) and measuring emission at 460 nm (bandwidth = 20 nm). Titrations were performed in technical triplicates, twice on two different days. Two outliers were removed that showed fluorescence signal that was more than twice the fluorescence intensity of the second highest value. All other values were plotted with the normalised mean fluorescence intensity (from N=6 and N=5) and standard error (calculated from N-1 method) using GraphPad Prism 7.0.

Titration experiments with high hpDNA concentrations were performed on a JASCO FP-8600 fluorimeter in 50 µL fluorescence cuvettes (Hellma Analytics).

UV/Vis spectra were recorded with a Jasco spectrophotometer V-770 using a 1 cm quartz cuvette. DMSO stocks were diluted in PBS to a final concentration of 20 µM or 10 µM in presence of 1 mM hpDNA. Data was plotted using Graphpad Prism 8.0.

Fluorescence spectra in the presence of 1 mM hpDNA were recorded on a JASCO FP-8600 fluorimeter in 50 µL fluorescence cuvettes (Hellma Analytics). Emission spectra were collected from 370 to 800 nm (bandwidth 10 nm) exciting at 350 nm (bandwidth 10 nm).

Quantum yields were determined using a Hamamatsu Quantaurus QY according to the manufacturer information in triplicates.

4.2. Cell culture

T-REx^{TM}-HeLa cells were cultured in high-glucose phenol red containing Dulbecco's modified Eagle's medium (DMEM, Life Technologies) supplemented with GlutaMAX (Life Technologies), sodium pyruvate (Life Technologies) and 10% heat-inactivated fetal calf serum (FCS, Life Technologies) in a humidified 5% CO_2 incubator at 37 °C. Cells were split every 2-3 days in a 1:5 ratio using Gibco™ Trypsin-EDTA (0.25 %, Life Technologies). The supernatant was regularly tested for mycoplasma contamination.

For seeding, T-REx^{TM}-HeLa cells were trypsinized and counted with a Countess II Automated Cell Counter (Thermo Fisher) in a bright field view at 1 x 10^5 to 1 x 10^6 cells/mL density. The cells were diluted to a final concentration of 2 x 10^5 cells/mL and seeded in a Greiner Bio-One CELLview™ Cell Culture Slide (10 wells, 20,000 cells/well, 100 µL culture medium).

Microscopy experiments were performed on a Leica DMi8 microscope (Leica Microsystems) equipped with a Leica TCS SP8 X scanhead, a SuperK white light laser, a UV CW laser (Coherent, 355 nm, 80 mW) and a heating chamber (37 °C and 5% CO_2, Life Imaging Services). The measurements were performed with a HC PL APO 63 ×/1.47 oil objective or a HC PL APO 40.0 ×/1.10 water objective.

4.3. HeLa cell experiments

HeLa cells were incubated with 0.1-10 μM pcHoechst 2-4 hours prior to the experiments. For global uncaging experiments the cells were irradiated with laser light at 355 nm (20-80%) for 10-15 s. The increase of the fluorescence signal was observed at 355 nm (2-5%) for 10 min with imaging one frame per minute. For long-term imaging or with imaging with a higher frame rate the data was acquired at 405 nm (2%).

For local uncaging of a single cell or a subnuclear fraction the bleachpoint function or region of interest (ROI) function of the FRAP module was used. Laser intensity: 2-80%; λ = 355 nm.

To study the cytosolic background, co-localization experiments with the endsomal / lysosomal system of HeLa cells were performed. HeLa cells were seeded in a low-volume 20 well ibidi dish (5000 cells/well) and allowed to settle and attach overnight. The cells were pre-incubated with mCLING-ATTO 647N-labeled (25 uL, 2 nmol/mL) for 30 min before pcHoechst (25 μL, final concentration 10 μM) was added to stain endosomes. After 30 min the cells were washed with pcHoechst (10 μM in imaging medium, 3x) and imaged live 1-2 hours after pcHoechst was applied. For each frame pcHoechst was globally uncaged for 10 s with UV light (20%, 355 nm laser, 10 s) before imaging with a diode laser (λ_{ex} = 405 nm, 2%; λ_{em} = 430-530 nm) and a white light laser (WLL, λ_{ex} = 647 nm; λ_{em} = 667-717 nm). For co-localization with lysosomes, LysoTracker™ Red DND-99 was used. HeLa cells were seeded and pcHoechst (10 μM) was applied together with LysoTracker. We report two conditions:

1) Live cells were incubated with LysoTracker (100 nM) for 30 min before the cells were washed with imaging medium (3x) in 10 well Greiner dishes (20,000 cells/well). For each frame pcHoechst was gently globally uncaged for 10 s with UV light (20%, 355 nm laser, 10 s) before imaging with a diode laser (λ_{ex} = 405 nm, 2%; λ_{em} = 430-530 nm) and a white light laser (WLL, λ_{ex} = 577 nm; λ_{em} = 597-647 nm).

2) Live cells were incubated with 50 nM LysoTracker® Red DND-99 (Thermo Fisher Scientific) for 1.5 h with HeLa cells in 10 well CELLview™ Slides (Greiner). After exchanging the medium, the cells were imaged live using a HC PL APO CS2 63x/1.47 oil objective (NA 1.47) with $\lambda_{Ex/Em}$ = 405/420-520 nm for pcHoechst and $\lambda_{Ex/Em}$ = 579/599-699 nm. To avoid oversaturation of the nuclear signal, pcHoechst was not uncaged in course of the experiment.

Photobleaching of Hoechst33342 was studied labelling HeLa cells with a 1:10,000 dilution of a 10 mg/mL Hoechst33342 stock solution for 20 min. The cells were washed with imaging medium (3x) and imaged with the same laser settings that were used for uncaging experiments with pcHoechst. To determine the bleachpoint function, fluorescent microscope slides (chroma) were bleached and the bleachpoint plotted at different laser intensities. For each laser intensity experiments were performed with minimum of triplicates for both, cell experiments and for determining the bleachpoint function.

4.4. Calculating Cytosolic/nuclear fraction of pcHoechst

pcHoechst channel images of single cells were duplicated in FIJI and two ROIs were defined: the full image and the nucleus by hand. Both ROIs were measured for their integrated density before and after the nucleus was deleted from every image. Cytosolic signal was calculated by dividing the integrated density after by the integrated density before nucleus removal. Data was averaged and plotted in GraphPad Prism 8. A total of 3 cells stained with pcHoechst from 3 different uncaging conditions were analyzed (n=9).

4.5. Calculating Pearson's R coefficient

Both pcHoechst and Lystracker channel images of single cells were duplicated in FIJI and two ROIs were defined: the full image and the nucleus by hand, the latter of which was deleted for further analysis. Image stack was split and Coloc2 analysis was applied on both channels with Costes' threshold regression. Pearson's colocalization coefficients above threshold were averaged and plotted in GraphPad Prism 8. A total of 6 cells stained with pcHoechst/LysoTracker were analyzed. The same 6 images served as negative control by rotating one channel around 180° and performing the same analysis. No bleedthrough was detected (*cf.* Figure S6, S7).

4.6. Zebrafish strains and maintenance

Zebrafish (*Danio rerio*) were maintained according to the guidelines of the local authorities under licenses GZ:565304/2014/6 and GZ:534619/2014/4. Zebrafish WT AB* (ZFIN ID: ZDB-GENO-960809-7) embryos were used for all *in vivo* experiments. All embryos were kept in E3 medium (0.63 g/l KCl, 14.0 g/l NaCl, 1.83 g/l $CaCl_2 * 2 H_2O$, 1.99 g/l $MgSO_4 * 7 H_2O$, pH 7.4) containing 0.003% Phenylthiourea (PTU, Sigma-Aldrich, St. Louis, MO) after 22 hpf to inhibit melanogenesis.

4.7. Determining (pc)Hoechst33342 toxicity and pcHoechst leakiness in zebrafish

For determining pcHoechst toxicity and leakiness, zebrafish embryos were dechorionated after 24 hpf and embedded in 1.2% low-melting agarose (Agarose Type IX-A, Ultra-low Gelling Temperature, Sigma-Aldrich) dissolved in E3 medium on micro-slides (µ-slide 4 well, Ibidi, Gräfelfing, Germany). Subsequently, treatment with 1% DMSO (vehicle control) 100 µM pcHoechst and 10 µM or 100 µM Hoechst33342 (H3570, Molecular Probes, Eugene, OR) was performed on mounted zebrafish embryos with the compounds mixed into 500 µl E3 medium containing 0.003% PTU and 0.3 mg/ml Tricaine (Ethyl 3-aminobenzoate methanesulfonate 98%, Sigma-Aldrich) layered on top of the agarose. For the incubation period, pcHoechst treated embryos were kept either in the dark by wrapping the micro-slides in aluminum foil or under ambient room light. UV-LED (395 nm) illumination was carried out at 32 hpf for 5 minutes. Images for pcHoechst toxicity were recorded at 52 hpf using a microscope camera (MC170 HD, Leica Microsystems, Wetzlar, Germany) mounted on a Leica M125 stereomicroscope and the LAS V4 software.

4.8. Determining uncaging toxicity

10,000 HeLa cells were seeded in 8-well Ibidi dishes and allowed to attach over night. Treatment was then performed with vehicle alone, 0.1 and 10 uM Hoechst33342 or 10 uM pcHoechst and cells were transferred without washing to an Nikon TI Eclipse microscope equipped with an automated stage (Märzhäuser Tango2), a multicolor LED excitation lamp (pE4000 CoolLED), DAPI/FITC/Cy3/Cy5/Cy7 Penta LED HC Filter Set (F66-615, AHF), a 20x Plan Apo NA0.75 objective (Nikon), a sCMOS camera (Neo, Andor) and equipped with a chamber for 37 °C and CO2 supply (Okolab). Cells were brought into focus in brightfield mode and a first image was acquired using Dapi Filter Set (200 ms exp. time, 365 nm Exc, Penta LED HC Filter Set, BP447/60 Em (Semrock)). Uncaging was then performed for 0, 4, or 10 seconds using using Dapi Filter Set (see intensity below) and a second DAPI image was acquired. Cells were then imaged for the next 24 h in phase contrast mode every 30 minutes.

After the timelapse, videos were evaluated blindly for successful cell division by manual counting. We defined such an event when a cell divided in 2 living cells. Numbers were pooled from two different regions of interest and are summarized below.

**counted cells**	**no UV**	**4 s UV**	**10 s UV**
mock	97	265	256
0.1 uM Hoechst	152	409	417
10 uM Hoechst	227	438	403
10 uM pcHoechst	152	425	444
**underwent division**	**no UV**	**4 s UV**	**10 s UV**
mock	74	104	33
0.1 uM Hoechst	61	39	8
10 uM Hoechst	2	0	0
10 uM pcHoechst	128	91	23
**division/counted in %**	**no UV**	**4 s UV**	**10 s UV**
mock	76.3	39.2	12.9
0.1 uM Hoechst	40.1	9.5	1.9
10 uM Hoechst	0.9	0.0	0.0
10 uM pcHoechst	84.2	21.4	5.2

4.9. mRNA microinjections

For pcHoechst uncaging experiments, zebrafish embryos were injected at the one-cell stage with an injection mix containing 25 ng/µl mRNA synthesized from pCS2+_H2B-mRFP plasmid using mMESSAGE mMACHINE™ SP6 Transcription Kit (Thermo Fisher Scientific, Waltham, MA). Injections were performed with glass capillaries pulled with a needle puller (P97, Sutter Instruments, Novato, CA), mounted onto a micromanipulator (World Precision Instruments Inc., Berlin, Germany) and connected to a microinjector (FemtoJet i4, Eppendorf, Hamburg, Germany).

4.10. *In vivo* UV laser-based single cell uncaging

Zebrafish embryos were dechorionated and embedded in 1.2% low-melting agarose on micro-slides at 32 or 56 hpf as previously described[34] and incubated with 100 µM pcHoechst or 10 µM Hoechst33342 for at least four hours. For the incubation period, embryos were kept in the dark by wrapping the micro-slides in aluminum foil. *In vivo* activation of pcHoechst was performed using the 405 nm UV laser of a confocal microscope (Leica SP8 WLL, Leica Microsystems, Wetzlar, Germany). The respective laser power was determined using a laser power meter (LP1, Sanwa, Tokyo, Japan). The bleachpoint function of the Leica LAS software was employed to target nuclei of cells in live zebrafish for uncaging. 100 ms light-pulses of 405 nm UV light every 3 seconds were used for illumination of the target site, repeated for 60-100 cycles. All photoactivation experiments at the confocal microscope were conducted at 28 °C using a temperature control system (The Cube 2, Live Imaging Services, Basel, Switzerland). Confocal microscopy images were recorded using the LAS X software (Leica Microsystems, Wetzlar, Germany), videos were edited and labeled with ImageJ.

4.11. Laser power and intensity calculations

In cellulo experiments (EPI-TIRF):

Laser powers were measured with a 20x air objective (NA 1.5) using a Thorlabs PM100A power meter (Newton, New Jersey, USA) collecting at 365 nm with an output power of the LED of 76 μW. The illuminated area using the radius r = 0.75 mm calculates to be 1.767 mm^2. The following table shows LED intensity, which was achieved in the illuminated area in the *in cellulo* experiments.

**% 365 nm LED**	**measured power (μW)**	**calculated intensity (mW/cm²)**
100	76	4.301

In cellulo experiments (Leica SP8):

Laser powers were measured with a HC PL APO CS2 63x oil objective (NA 1.47) using a Thorlabs S170C microscope slide power meter sensor (Newton, New Jersey, USA) collecting between 30 and 50 data points with a SuperK white light CW laser (Coherent) at 355 nm with an output power of the laser of 65 mW and different laser settings. Using a 63x objective the beam diameter was calculated using the following formula: 1.22 × 355 nm / 1.47 = 295 nm and the illuminated area using the bleachpoint function is 86,804 nm^2. The following table shows lasers intensities, which were achieved in the illuminated area then applying different laser powers in the *in cellulo* experiments.

**% 355 nm laser**	**measured power (μW)**	**calculated intensity (W/cm²)**
100	1,260 ± 26.4	1,451,546
80	1,100 ± 25.1	1,267,223
60	777 ± 15.8	895,120
40	406 ± 10.7	467,720
20	110 ± 10.7	126,722
10	28,2 ± 3.60	32,487
5	7.16 ± 0.247	8,248
2	1.42 ± 0.127	1,636
0	0.102 ± 0.236	118

In cellulo experiments (Zeiss LSM 880 for 2 photon uncaging):

2 Photon microscopy experiments were performed on a Zeiss LSM 880 AxioExaminer Z1 upright microscope equipped with a Coherent Chameleon II NLO laser using ZEN Black 3.2 software. The measurements were performed with a Plan-Apochromat 20x/1.0 water dipping objective, an excitation at 720 nm and emission detected with an PMT at 420-520 nm with an open pinhole and a pixel size of 0.15 µm.
Photoactivation was achieved with 720 nm excitation with 50% laser power in a round ROI of 20 pixel with 15 scan iteration and a pixel dwell time of 1.64 µs.
Laser powers were measured with a Plan-Apochromat 20x water dipping objective (NA = 1.0) using a Coherent LabMax TO power meter with an LM10 sensor at 720 nm with a laser output power in the ZEN Software in % (see table below) with a MBs 690+, continuous scan.
Intensities were calculated as follows:

$$I = (4 * \ln 2 * P_{measured}) / (\pi * (0.52 * 720 \text{ nm} / NA)^2) =$$
$$= (2.772 * P_{measured}) / (\pi * (0.52 * 720 \text{ nm} / 1.0)^2) =$$
$$= (2.772 * P_{measured}) / 440{,}374 \text{ nm}^2 =$$
$$= 6.295 * 10^8 P_{measured} \text{ cm}^{-2}$$

**% 720 nm laser**	**measured power (mW)**	**calculated intensity (W/cm²)**
50	19.7	12,403,094
10	5.6	3,525,752
5	2.5	1,573,997
0.2	0	0

In vivo experiments:

Laser powers were measured with a 40x water objective (NA 1.1) using a Nova II power meter (Ophir, North Logan, USA). Using a 40x objective the beam diameter was calculated using the following formula: 1.22 x 405 nm / 1.1 = 449 nm and the illuminated area using the bleachpoint function is 158,478 nm^2. The following table shows lasers intensities, which were achieved in the illuminated area then applying different laser powers in the *in vivo* experiments.

**% UV laser**	**measured power (mW)**	**calculated intensity (W/cm²)**
100	0.24	151,440
60	0.20	126,200
20	0.07	44,170
5	0.0175	11,043

5. Supplemental Figures

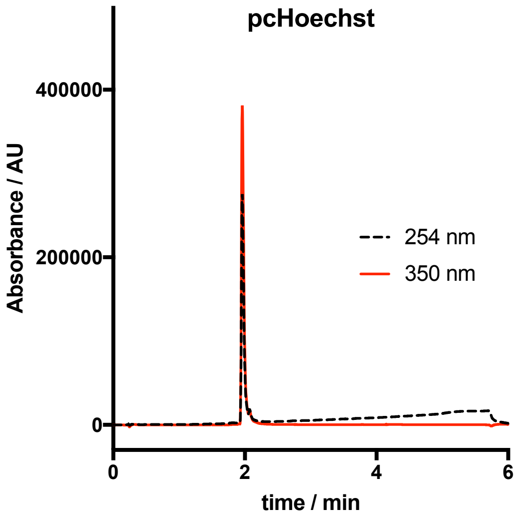

Figure S1: UHPLC trace of pcHoechst with absorbance at 254 nm (black) and 350 nm (red). Peak AUC measurement gives purity >95%.

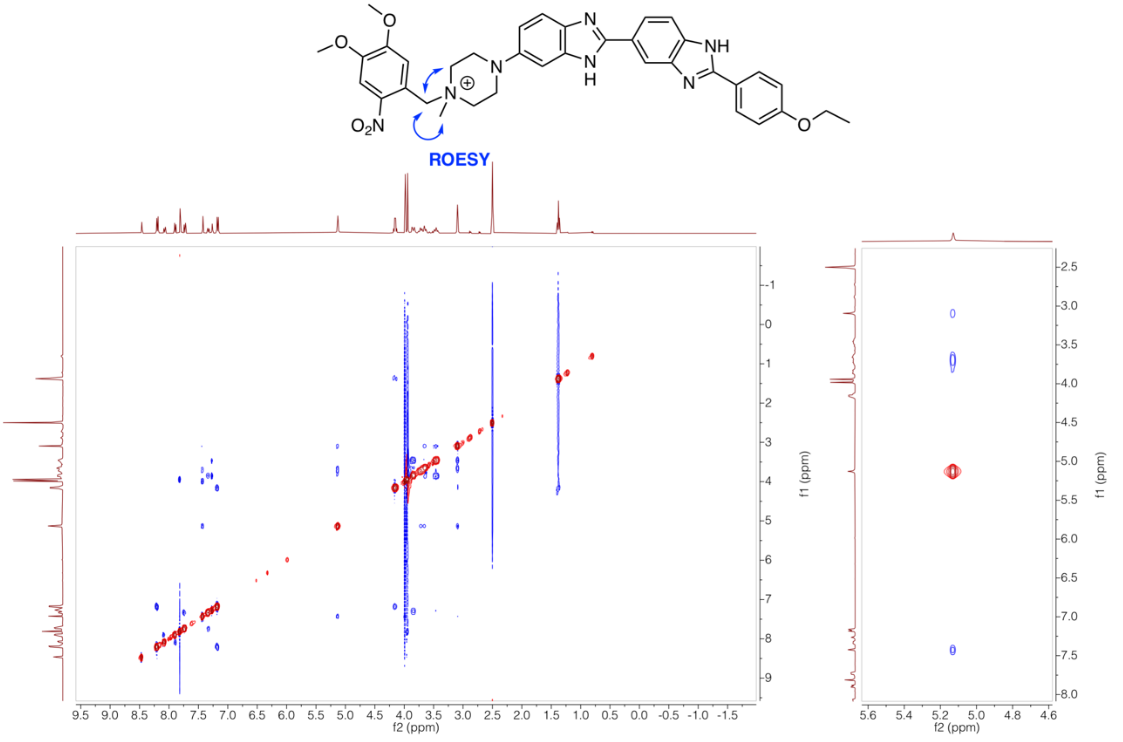

Figure S2: ^{1}H-^{1}H ROESY shows coupling of the benzylic protons to the methyl and methylene group of the piperazine, proving the regiochemistry of pcHoechst.

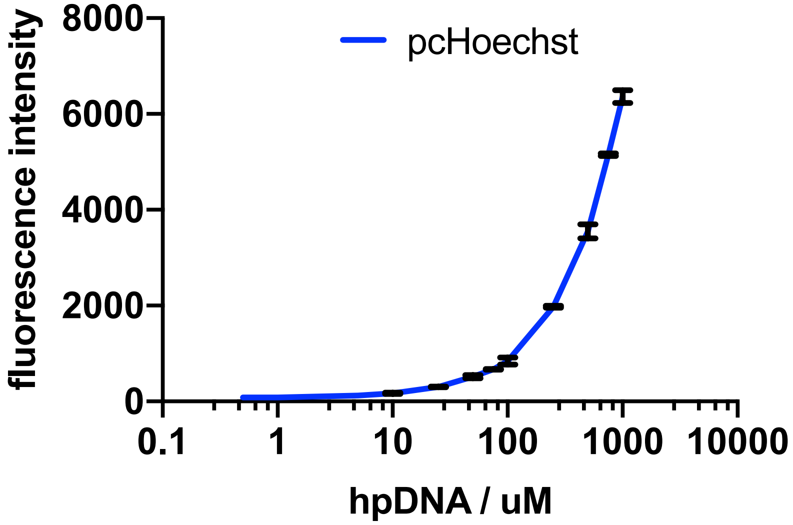

Figure S3: Extended pcHoechst titration shows increase in fluorescence at high hpDNA concentrations.

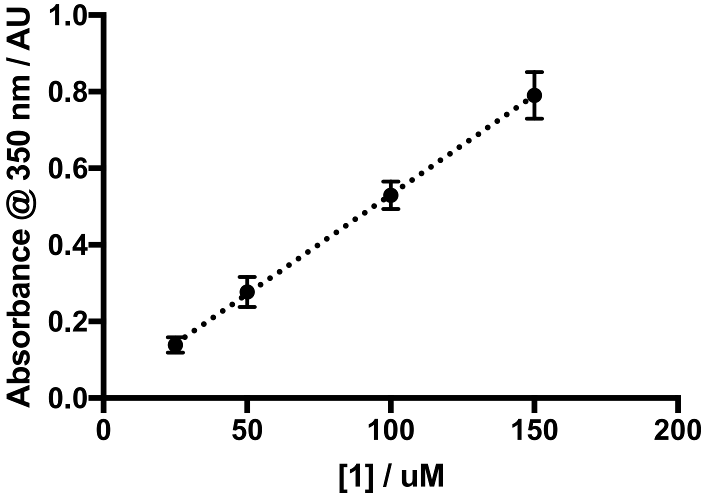

Figure S4: Determination of the extinction coefficient at 350 nm of **1** in PBS according to Lambert-Beer. $\varepsilon_{350\ nm}$ (**1**) = 5187±47 M^{-1} cm^{-1}

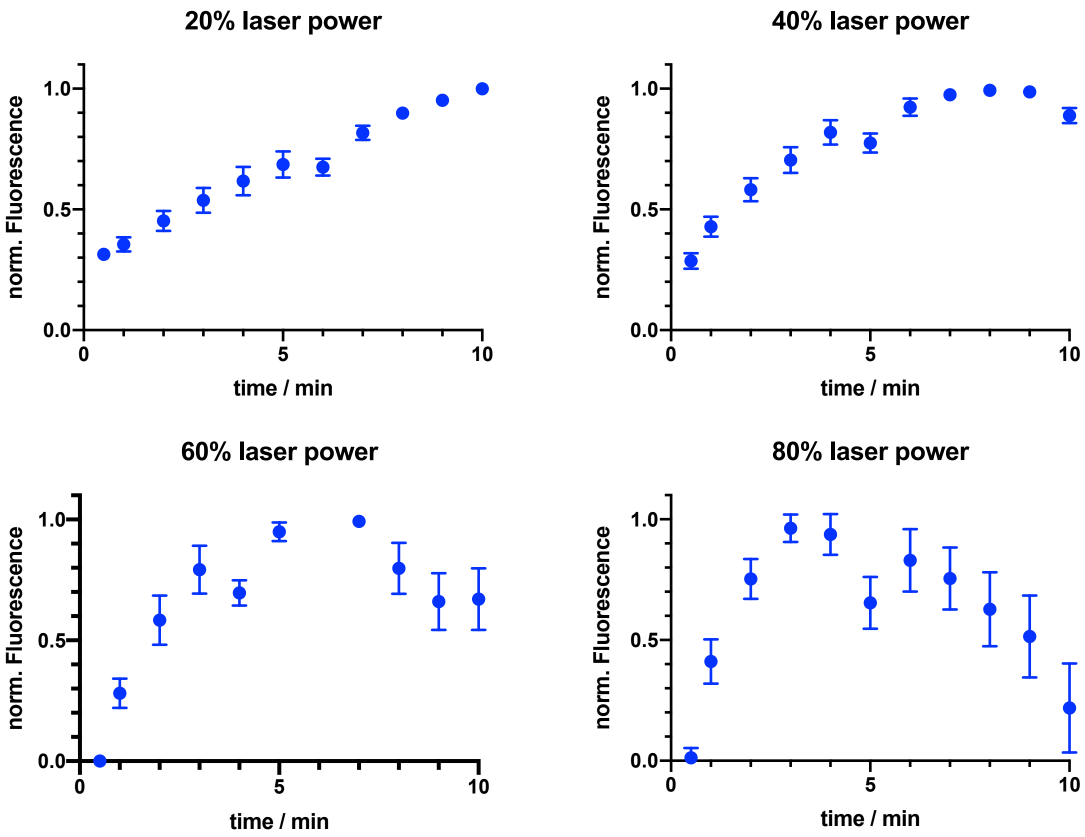

Figure S5: Laser power intensity on global uncaging of pcHoechst in HeLa cells. Laser powers between 20–80% were used to uncage pcHoechst in live HeLa cells and following fluorescence increase in nuclei. While a slower increase could be observed at lower intensities, 60% laser power reached saturation within 5 minutes, and longer irradiation led to a signal decrease, presumably to bleaching, which is supported by faster decreasing rates using 80% laser power. n>12 cells.

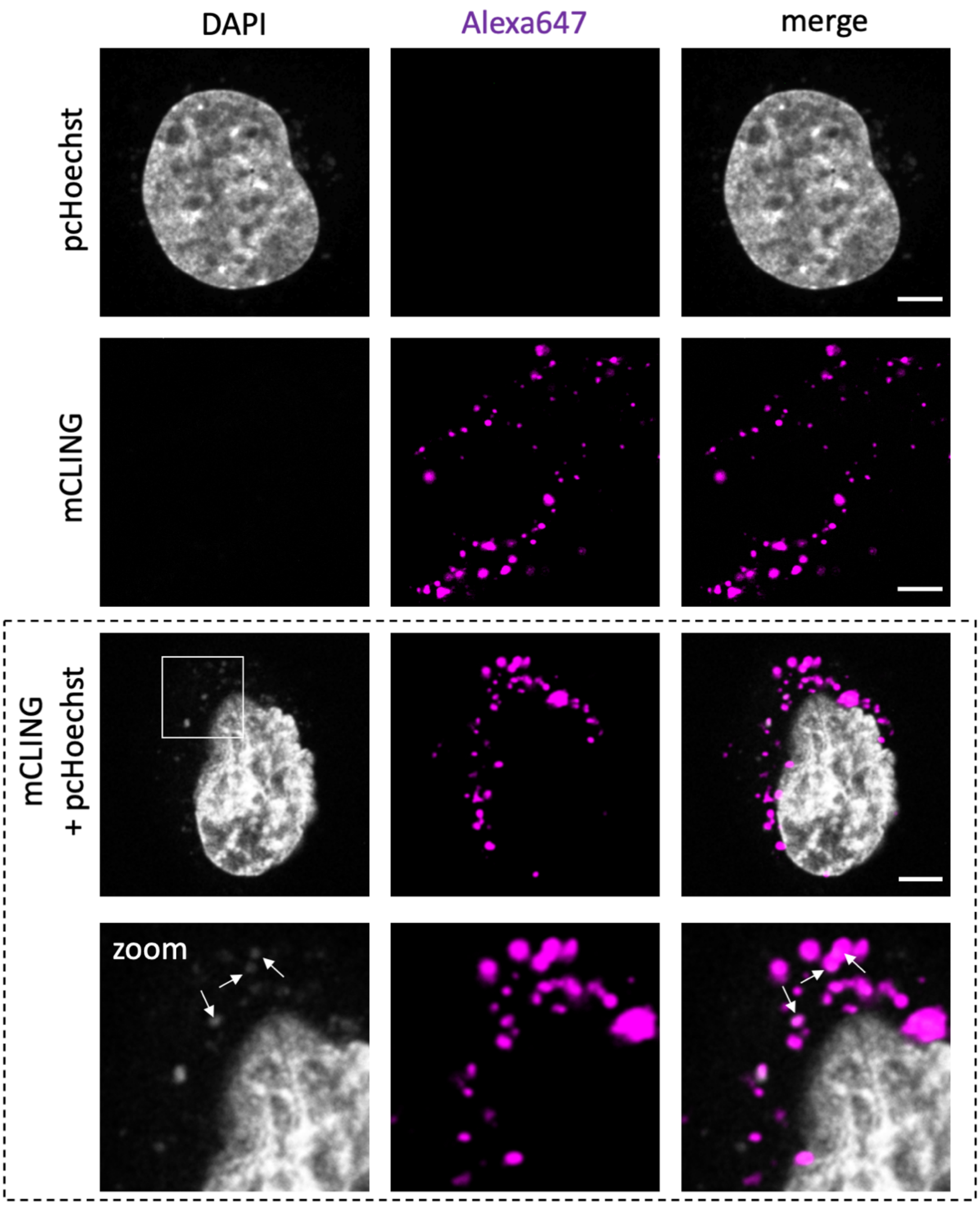

Figure S6: Colocalization of cytosolic signals from pcHoechst with the endo- and lysosomal system labelled mCLING. Scale bar = 5 µm.

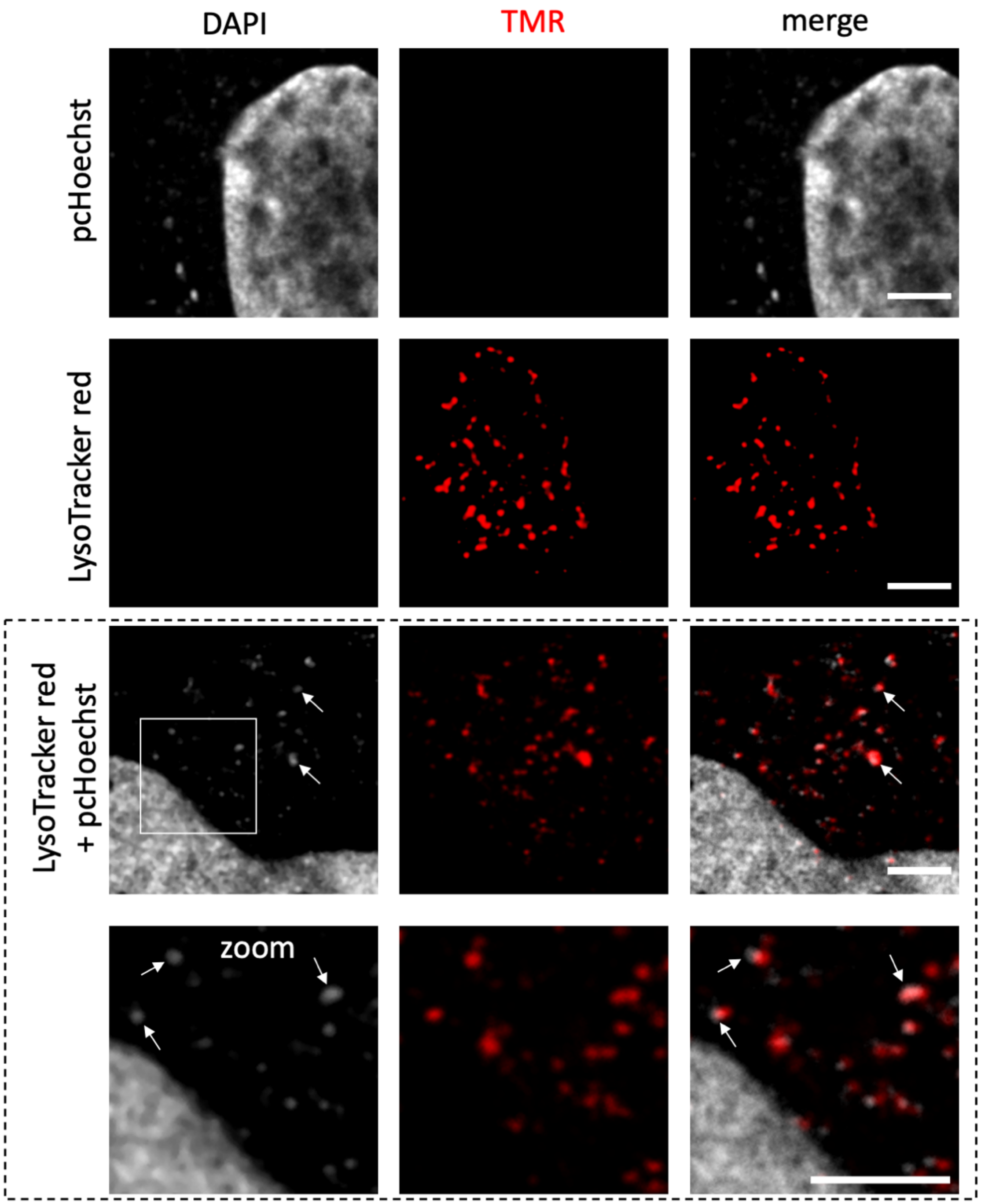

Figure S7: Colocalization of cytosolic signals from pcHoechst with the lysosomal system labelled LysoTracker™ red. Scale bar = 5 µm.

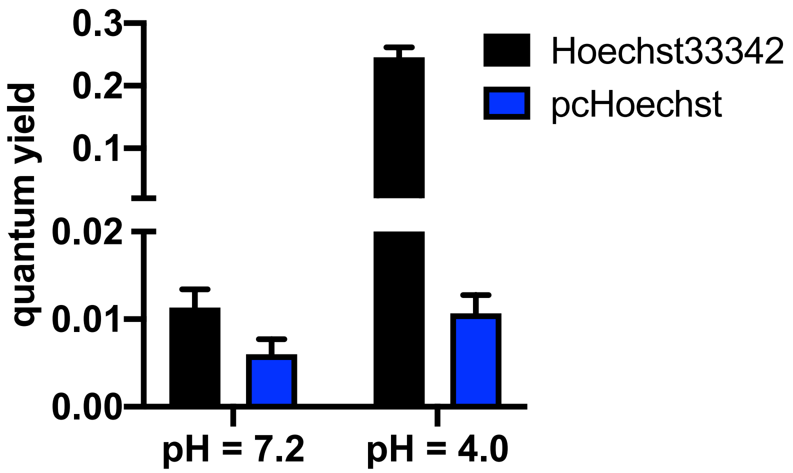

Figure S8: Hoechst33342 shows marked quantum yield increase in citric acid buffer (pH = 4) vs. PBS (pH = 7.2), while pcHoechst quantum yield only increases slightly.

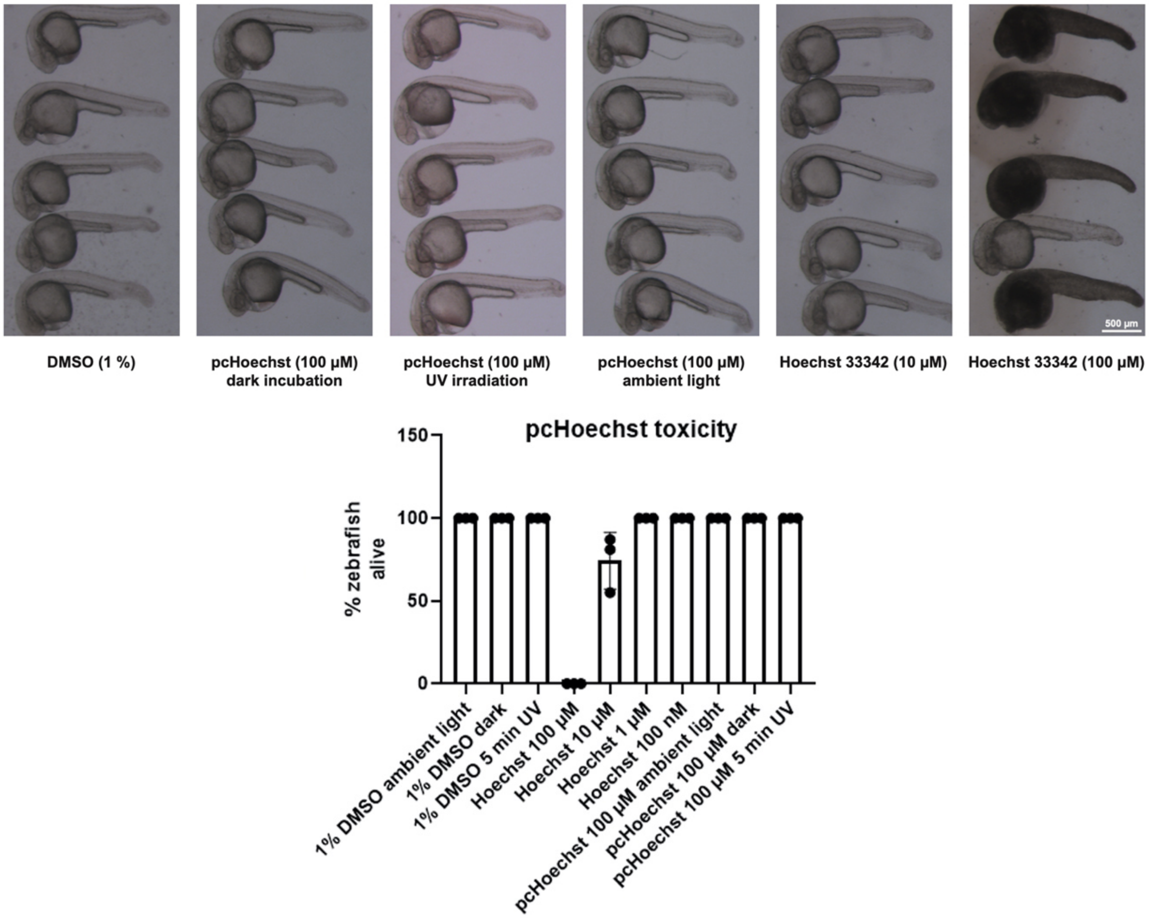

Figure S9: pcHoechst is not toxic at high concentrations to zebrafish embryos. Toxicity of 100 μM pcHoechst was assessed under various conditions (dark, ambient light, 5 min UV-LED illumination at 395nm) and compared to 1% DMSO (dark, ambient light, 5 min UV-LED illumination at 395nm), 100 nM, 1, 10 and 100 μM Hoechst 33342. Whereas pcHoechst did not show toxicity under any condition in embryos upon incubation from 24 hpf to 48 hpf, Hoechst33342 showed some toxicity at 10 μM and was lethal at 100 μM. Representative images for the respective condition are shown on the upper panel. The bar plot shows mean % of zebrafish embryos alive (n = 3 experiments with a total of 49-76 zebrafish per condition). Scale bar is 500 μm.

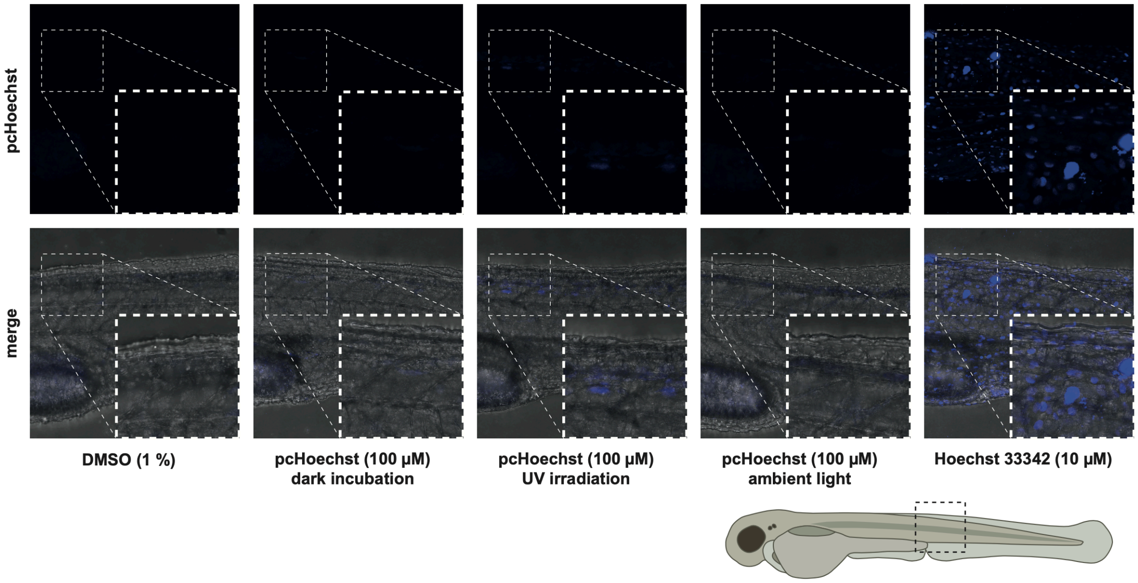

Figure S10: Leakiness of pcHoechst in the dark or under ambient light. Zebrafish embryos were incubated in 100 µM pcHoechst from 26 hpf to 54 hpf either in the dark, under ambient light or in the dark with a 5 min UV-LED (395 nm illumination at 32 hpf). Control embryos were incubated in 1% DMSO (negative control) or in 10 µM Hoechst 33342 (positive control). For pcHoechst, blue fluorescence was only observed upon UV-illumination, indicating that the molecule is stable under ambient light. Representative images were recorded on a confocal microscope (Leica SP8) using a 10x objective. n = 3

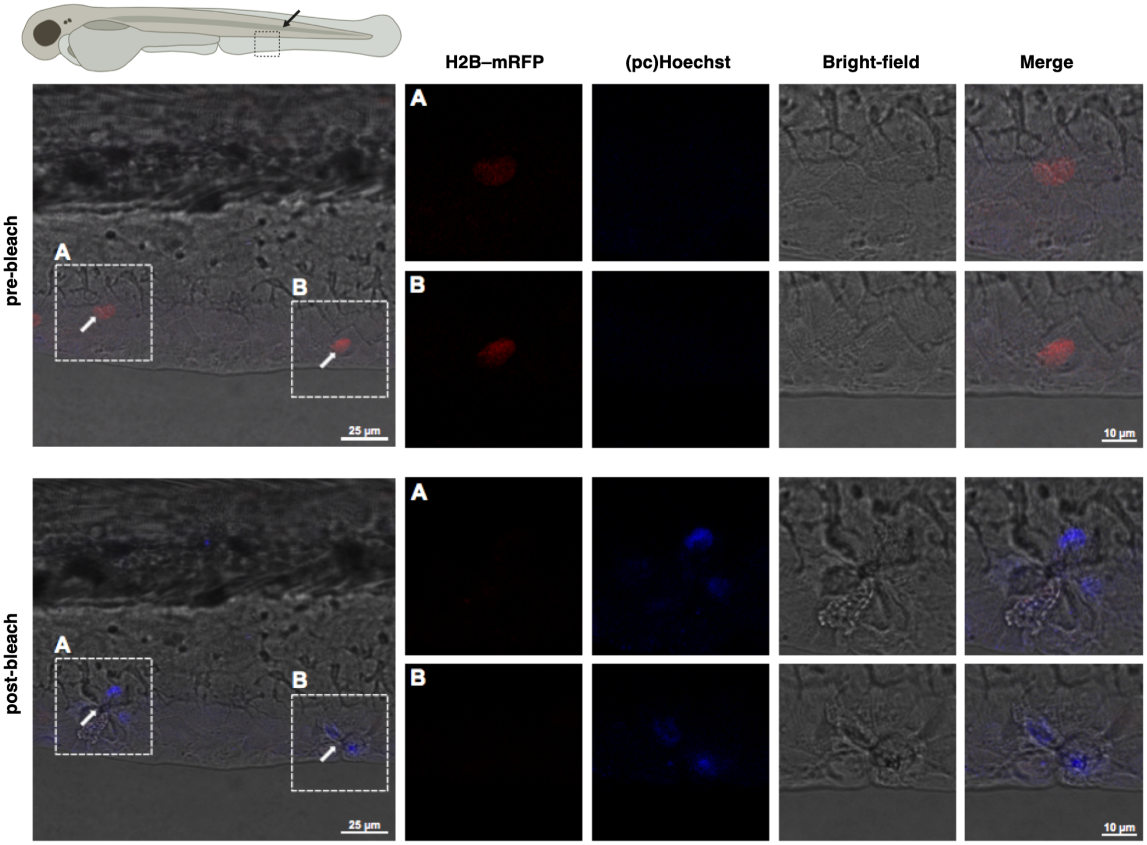

Figure S11: Spatially confined uncaging of pcHoechst *in vivo*. H2B-mRFP (red) injected zebrafish embryos were incubated with 100 µM pcHoechst in the dark overnight and activated using a 405 nm UV-laser at 0.2 mW at 72 hpf. Arrows indicate nuclei targeted with two bleachpoints each. Pre-bleach image (upper row) was recorded prior to bleach-point illumination, post bleach image (lower row) 15 min after bleaching. A and B are magnified areas as indicated by the white boxes. mRFP-tagged protein can be observed before UV illumination and is subsequently bleached, while the uncaged pcHoechst (blue) signal can only be observed after the bleaching step. Merge includes bright-field image. Scale bars are: overview image 25 µm, zoomed-in 10 µm.

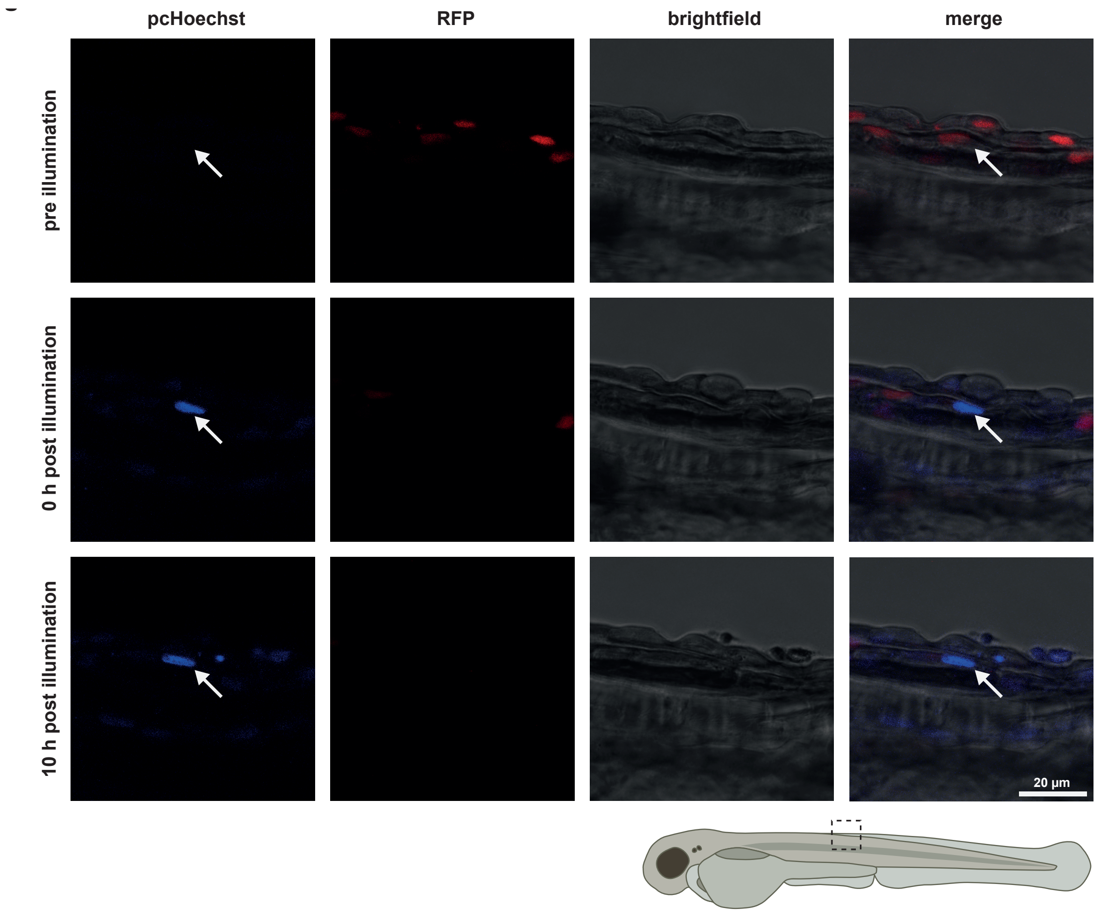

Figure S12: Uncaged pcHoechst can be detected for multiple hours post UV illumination. Zebrafish embryos were microinjected with pCS_H2B-mRFP mRNA, mounted on micro-slides at 32 hpf and incubated with 100 µM pcHoechst in the dark overnight. 405 nm UV laser illumination was performed using the bleachpoint function at an intensity of 0.2 mW every three seconds for 60 cycles at 56 hpf. Arrow points at the position of a nucleus labeled by uncaged pcHoechst (blue) after illumination (upper row) and 10 h post illumination (lower low). Images are taken from a time-lapse movie recorded on a Leica SP8 confocal microscope using a 40x objective.

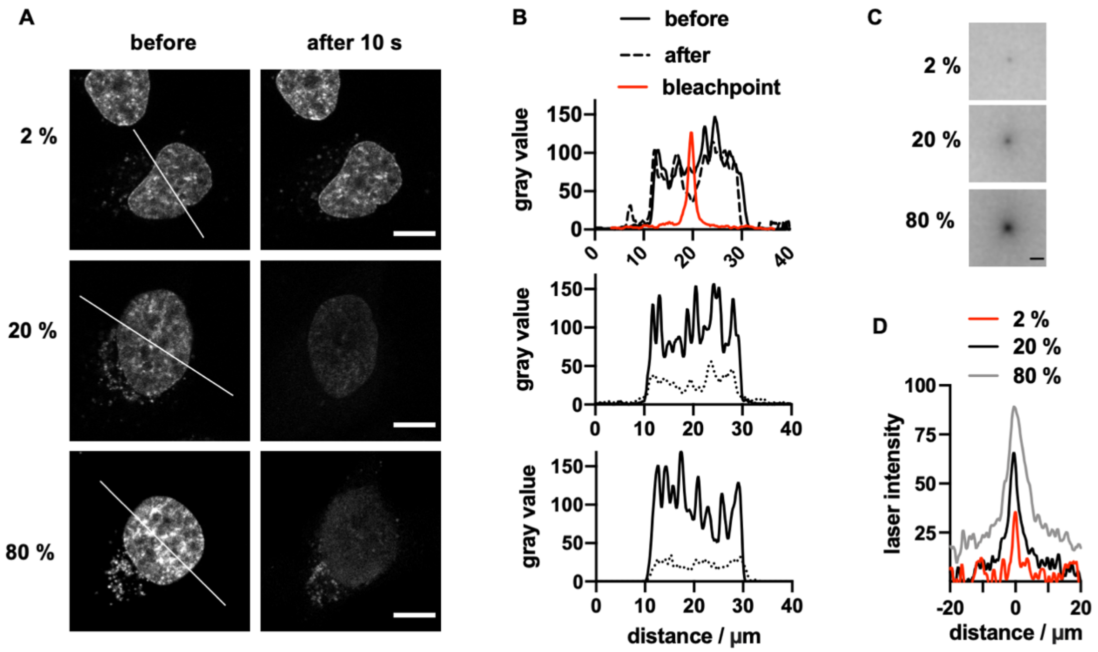

Figure S13: Photobleaching of Hoechst33342. A) Confocal images before and after irradiation of live HeLa cells labelled with Hoechst33342 with UV light using a bleachpoint function at different laser intensities for 10 s. Scale bar = 10 µm. B) Line profile of HeLa cells from (A) before and after bleaching. C) Bleachpoints on a fluorescent coverslip with (D) plotted peak profiles. Scale bar = 10 µm.

6. Supplemental Movies

Supplemental Movie 1–4: EPI-TIRF imaging. HeLa cells were incubated with 10 µM compound and ± UV light:
Supplemental Movie 1: 10 µM Hoechst, no UV
Supplemental Movie 2: 10 µM Hoechst, + UV
Supplemental Movie 3: 10 µM pcHoechst, no UV
Supplemental Movie 4: 10 µM pcHoechst, + UV
Image acquisition was in brightfield mode every 30 minutes for 24 h.
Supplemental Movie 5: Confocal imaging: HeLa cells were incubated with 10 µM pcHoechst for 2 h and then globally uncaged using UV light (λ_{uncage} = 355 nm, 15 sec, 20% laser intensity, line scans) and followed by image acquisition ($\lambda_{Ex/Em}$ = 405/450-500 nm) every 30 sec. The first frame is recorded directly after uncaging.
Supplemental Movie 6: Confocal imaging: Zebrafish imaging was performed for 1.5 hours post-uncaging.
Supplemental Movie 7: Confocal imaging: HeLa cells were incubated with 10 µM pcHoechst for 4 h and then globally imaged and bleached using 2 photons (λ = 720 nm, 10%, line scans).

7. Reference

[1] G. Lukinavičius, C. Blaukopf, E. Pershagen, A. Schena, L. Reymond, E. Derivery, M. Gonzalez-Gaitan, E. D'Este, S. W. Hell, D. W. Gerlich, et al., *Nat. Commun.* **2015**, *6*, 1–7.

[2] P. E. Pjura, K. Grzeskowiak, R. E. Dickerson, *J. Mol. Biol.* **1987**, *197*, 257–271.
